# Exploring the unique and cumulative effects of individual-level and social determinants on suicidal ideation trajectories during health and environmental crises – a longitudinal study of Australians

**DOI:** 10.1186/s12889-026-26816-4

**Published:** 2026-03-02

**Authors:** Quincy J. J. Wong, Sandersan Onie, Lauren McGillivray, Alexander Burnett, Adam Theobald, Fiona Shand, Michelle Torok, Mark E. Larsen

**Affiliations:** 1 School of Psychology, Western Sydney University, Sydney, Australia; 2 Black Dog Institute, University of New South Wales, Sydney, 2031 Australia; 3 Centre for Big Data Research in Health, University of New South Wales, Sydney, Australia

**Keywords:** Suicidal ideation, Suicide, Risk factors, Social determinants, Longitudinal study, Trajectory

## Abstract

**Background:**

There is a paucity of research that has examined how significant health, social, and environmental crises impact suicidal ideation, particularly where these events co-occur and have potentially synergistic adverse effects. The aim of this study was to identify the unique and cumulative contributions of mental health, social, and environmental risks on 12-month trajectories of suicidal ideation.

**Methods:**

A community-based sample of 1928 Australians (aged ≥ 16 years) completed online surveys measuring mental health, social, and environmental risk factors, and suicidal ideation at baseline, and the suicidal ideation measure was repeated at four follow-up timepoints. Growth mixture modelling identified suicidal ideation trajectories and multinomial logistic regression analyses examined associations between baseline risk variables and the trajectories.

**Results:**

Analyses showed five different trajectories of suicidal ideation (one stable low ideation trajectory; four vulnerable trajectories). A different set of risk factor variables predicted each vulnerable trajectory relative to the low trajectory. A cumulative risk index representing the combined effects of the mental health, social, and environmental risk factors also predicted each vulnerable trajectory relative to the low trajectory.

**Conclusions:**

While adversity resulting from exposure to health and environmental crises was associated with suicidal ideation, the heterogeneity in how patterns of mental health, social, and environmental factors related to trajectory classes suggests that people experience these events differently. A cumulative risk index may be a potentially useful indicator of risk.

**Supplementary Information:**

The online version contains supplementary material available at 10.1186/s12889-026-26816-4.

## Background

Suicide is a major global concern, accounting for approximately 700,000 deaths each year [1]. Suicidal ideation is an important precursor to suicide, increasing the risk of suicide attempt [2] and suicide [3], and as such, is an important target for public health prevention efforts. The factors contributing risk to the onset and maintenance of suicidal ideation are often complex, occurring at the individual, relational, community, and societal levels [4]. Understanding how these factors influence patterns of suicidal ideation over time have potential to shape national, whole-of-government responses to preventing suicide.

The social determinants of health are an important group of factors likely to influence trajectories of suicidal ideation. Social determinants reflect the conditions in which people learn, live, and work, and the systems that affect those conditions [5]. The COVID-19 pandemic brought to the fore a number of social determinants of relevance to mental health. In many countries, including Australia, restriction measures to prevent the spread of COVID-19 (e.g., stay at home orders) resulted in physical isolation, unemployment, reduced work hours due to workplaces shutting or changing their normal operations, and economic recession as various industries and businesses halted (e.g. [6]), . Many countries enacted financial support initiatives to counter negative impacts of these economic consequences on suicide and mental ill-health [7]. While some of these factors have been individually linked to suicide risk, such as isolation, financial strain, and serious illness (e.g. [8, 9]), their co-occurrence is rare, making the pandemic context a unique opportunity to concurrently examine the impact of these factors.

The restriction measures had other adverse effects that have been associated with an increased risk of suicide. For example, an involuntary increased amount of time with household members was associated with an increased rate of domestic violence [10, 11]. The loss of employment or changes in work resulting in reduced income meant that many were not able to pay their mortgage or rent, with evidence that the lockdowns were associated with housing security concerns [12]. With these impacts and the associated stress apparent, there was also evidence of increases in alcohol and substance use [13].

Although the multiple impacts of COVID-19 are significant, at the same time of the pandemic, Australians were exposed to multiple natural disasters [14, 15]. Following a period of drought, and 2019 being the hottest and driest year on record for Australia [16], the 2019/20 bushfire season resulted in over 19 million hectares burnt, over 3,000 houses destroyed, and 33 people killed [17]. This was followed by heavy rainfall in New South Wales in early 2020, which caused major flooding [16]. With exposure to natural disasters being a significant risk for suicide, the potential for significant economic and psychological cumulative impacts of these health and environmental events are important to consider [18, 19].

There are a limited number of studies that have examined longer term patterns of suicidal ideation alongside significant health, social, and environmental crises. Several longitudinal studies of adults found no significant changes in the overall prevalence of suicidal ideation during different periods of the COVID-19 pandemic [20–23] while others showed an increased prevalence [24, 25], and one study showed a mixture of trends [26]. These studies showed that mental ill-health [20, 24], loneliness or being single [20, 21, 26], experiencing adverse events [20], particularly psychological or physical abuse [22], financial distress/adversity or being in a lower socioeconomic group [20, 22, 24], and disruptions to social and work domains [20] were all associated with suicidal ideation. However, most of this COVID-19 literature focused only on the effects of the pandemic, and the confounding or cumulative impact of parallel crises, and their co-occurring risks, have not been accounted for.

To our best knowledge, no studies have yet applied a ‘cumulative risk approach’ [27, 28] to assess the combined risk of health, social, and environmental factors on suicidal ideation trajectories. Moreover, the focus on changes in prevalence of suicidal ideation in previous COVID-19 literature means that there has been no investigation of the changes in the severity of ideation severity over time, which is important given that severe ideation may facilitate suicidal behaviour. With limitations of prior studies in mind, the current study aimed to test the potential for different trajectories of suicidal ideation severity across five timepoints during a period of significant health and environmental crisis in Australia, and to explore whether mental health, social, and environmental risk factors: (a) are uniquely associated with different suicidal ideation trajectories, and (b) can combine to form an index of cumulative risk that is associated with different suicidal ideation trajectories.

## Method

### Study design

This survey study used a 52-week prospective longitudinal design. The primary outcome was suicidal ideation measured at baseline (Week 0), and four follow-up timepoints (Weeks 4, 12, 26 and 52). This study examines risk variables measured at baseline on suicidal ideation trajectories.

### Participants

A convenience sample of 1928 community-based Australians were recruited online between 12 August and 24 September 2020 (during second wave of COVID-19 pandemic in Australia) via social media advertisements (see Results for sample characteristics). Eligibility criteria were: aged 16 years or older, residing in Australia, fluent in English, and having access to a computer or smartphone for completion of surveys. These criteria were assessed via a bespoke online screening tool hosted on Qualtrics, and individuals who were not eligible were provided with details of crisis support services. There were no specific exclusion criteria. Participants could opt into a prize draw to win one of 20 x $50 e-gift cards at each of the assessment timepoints.

## Measures

### Primary outcome

The 5-item Suicidal Ideation Attributes Scale (SIDAS) [29] was used to assess past-month suicidal ideation severity. Items are rated on an 11-point scale (0 to 10), with total scores ranging from 0 to 50, and higher total scores reflecting greater suicidal ideation severity. Scores ≥ 21 indicate high risk of suicidal behaviour [29]. The SIDAS has good reliability (e.g., Cronbach’s alpha = 0.87 in the current study) and validity [29].

### Risk variables

There were eight risk domains, each assessed with multiple variables to capture different aspects of a risk domain. Use of multiple variables for each domain ultimately enabled tests of whether specific aspects of risk domains were predictive in the analyses. Full details of risk variables are included in Supplementary File 1.

### Finance

There were four measures of different aspects of financial risk: participants’ net household income *before* COVID; net household *income changes* due to COVID-19; participants’ economic situation due to COVID-19; and whether participants sought financial support from the government.

### Housing

There were two measures of housing risk: whether participants owned a property at the time of the survey; and participants’ housing security due to COVID-19.

### Social isolation

There were four measures of social isolation risk. The first measure was the UCLA 3-item Loneliness Scale [30] with an additional item (*I have meaningful social contact*; reverse scored) included. The second measure was an adapted version of the Social Support Questionnaire [31] which had 6 hypothetical situations and produced two measures of social support: (a) whether participants had someone to help them in the situation (Yes/No) and (b) how dissatisfied participants were with the level of help. The number of ‘yes’ responses over the 6 situations was taken as a measure of social support and the average of the dissatisfaction ratings was taken as a measure of social support dissatisfaction. The fourth measure listed several community-based activities (e.g., volunteering, attending community organised events) and participants endorsed those they engaged in.

### Extreme weather and climate events (EWCEs)

There were three custom measures of participants’ EWCE-related risk. The first measure listed different EWCEs (e.g., *drought*, *bushfire*, *flood*) and participants endorsed those that they have personally experienced in the past 12 months. The second measure again listed different EWCEs and asked participants to endorse the EWCEs that have directly affected the region in which they lived in the past 12 months. The third measure listed potential effects of EWCEs (e.g., *My property was damaged/destroyed*, *I became injured*) and participants endorsed those that have happened to them.

### Employment

There were two measures of employment-related risk. The first measure reflected whether participants were employed at the time of the survey. The second measure reflected participants’ employment situation due to COVID-19.

### Interpersonal violence and conflict

There were two measures of risk related to interpersonal violence and conflict. The first measure was the 5-item Extended Hurt, Insult, Threaten, Scream (E-HITS) Screening Tool [32] which assessed interpersonal violence experienced in the past month. The second measure reflected the frequency of conflict, stress, or tension between members of participants’ household.

### Alcohol and substance use

A modified 4-item Tobacco, Alcohol, Prescription medications, and other Substance Self-administered Screen (TAPS-1) [33] was used to produce four measures of risk related to alcohol and substance use. The modified TAPS-1 asked participants about their use of tobacco, alcohol, illicit drugs and non-medical use of prescription medications in the past month.

### Mental health

There were four measures related to mental health. The first measure assessed whether participants had attempted suicide in their lifetime. The second measure assessed whether participants had engaged in non-suicidal self-injury in their lifetime. The third measure was the Patient Health Questionnaire – 8-item depression scale (PHQ-8) [34] which assessed depression symptoms in the past two weeks without assessing suicidal or self-injurious thoughts. The PHQ-8 was chosen for this study to prevent overlap with the primary outcome. The fourth measure was the Generalised Anxiety Disorder-7 (GAD-7) [35] which assessed anxiety symptoms in the past two weeks.

### Procedure

This study was approved by the University of New South Wales Human Research Ethics Committee (HC200321). Study adverts were posted on the Black Dog Institute website and social media accounts. Interested individuals were directed to the study’s website and provided with information about the study. After providing informed consent, participants completed screening and if eligible for the study were then asked to complete the online baseline survey. Participants were subsequently emailed a link to the online follow-up surveys at the required timepoint.

### Statistical analyses

Sample size determination was based on estimation accuracy in a growth mixture modelling framework. Simulation studies show that when separating 2–6 classes with five measurement timepoints, approximately 700–2000 individuals are required for accurate estimation in models, taking into account missing data and different levels of class separation [36]. Our sample size was therefore sufficient for models with up to 5 classes (see Supplementary File 2).

Trajectories of SIDAS scores were first examined. Across baseline and follow-ups, there was 4189 datapoints out of a possible 9640 datapoints (43.5% completion rate). Participants with missing SIDAS data significantly differed from those without missing data on some of the baseline variables (e.g., age, property ownership, variables related to alcohol and substance use; all *p*s < 0.036), indicating missingness could be explained by observed variables, consistent with a missing at random (MAR) assumption. As such, growth mixture modelling with full information maximum likelihood estimation under a MAR assumption and robust standard errors was used to classify participants into classes with similar SIDAS total score trajectories across the timepoints. Based on the shape of plotted mean SIDAS total scores across timepoints, initial analyses tested a one class linear model and a one class quadratic model. Factor loadings for the linear slope factor were set to 0, 0.04, 0.12, 0.26, and 0.52 reflecting the assessment timepoints, and these loadings were squared for the quadratic slope factor. Model testing proceeded with estimating models with increasing number of classes that yielded proper solutions. For models with multiple classes, means of latent variables (e.g., intercept and linear slope) were initially allowed to vary across classes, before allowing means and variances, and then means, variances, and covariances to vary across classes. Model fit was evaluated using the Bayesian Information Criterion (BIC; lower value = better model) and where appropriate the bootstrapped likelihood ratio test (BLRT; *p* value ≤ 0.05 indicates the model with more classes fits significantly better than the model with less classes). Selection of the optimal model was based on model fit and meaningfulness of the solution (e.g., number of individuals forming a class; whether emerged classes provided new insights). For the optimal model, to indicate classification quality, we report entropy (> 0.60 reflects good overall class separation [37]), and the average posterior probabilities for each class (> 0.70 reflects adequate accuracy for class assignment [38]).

Subsequent analyses examined associations between baseline risk variables and classes of the optimal model from the growth mixture modelling analyses. For the baseline risk variables, there were 44,023 datapoints out of a possible 48,200 datapoints (91.33% completion rate). Participants with missing baseline risk variable data significantly differed from those without missing data on some of the baseline variables (e.g., loneliness, depression; all *p*s < 0.001), suggesting missing data were plausibly MAR. Based on this, multiple imputation was used to handle missing data on the baseline risk variables (10 imputed datasets). The number of imputed datasets was based on the proportion of missing values [39]. All 25 risk variables and a suicidal ideation trajectory class variable that coded all classes were included in the multiple imputation model. All subsequent analyses conducted used the multiple imputation datasets. All subsequent analyses also used the suicidal ideation trajectory class variable as the dependent variable. In preliminary analyses, separate univariate multinomial logistic regression analyses were conducted for each baseline risk variable, to determine each variable’s associations with class. For the main analyses, because risk domains were assessed with multiple variables, and because the study aimed to determine unique associations (i.e., adjusted for other risk variables in the model), we focused on: (a) separate multivariate multinomial logistic regression analyses for each risk domain, to determine the variables within each risk domain which have unique significant associations with class, and then (b) a single final multivariate multinomial logistic regression analysis which simultaneously examined all variables (from all risk domains) with unique significant associations with class evident in previous analyses, to determine the variables across risk domains which have unique significant associations with class. Notably, in all multivariate models, the variance inflation factors (VIFs) for all risk variables were < 10, indicating multicollinearity was not a problem [40].

A cumulative risk index was then developed from the 25 baseline risk variables. A z-score approach based on Ettekal et al. [27] was used to develop the cumulative risk index, and this approach enabled risk to be conceptualised on a continuum and risk variables to differ in their contribution to the cumulative risk index. For this approach, all baseline risk variables (continuous and categorical) were scaled such that higher scores indicated greater risk, z-scores were then calculated for each variable, and an average of these z-scores was subsequently calculated for each participant to produce the cumulative risk index. Separate univariate multinomial logistic regression analyses were used to test whether the cumulative risk index was associated with class membership based on the emerged classes from the growth mixture modelling. In all multinomial logistic regression analyses, relative risk ratios (RRR) were reported as a measure of association with *p*-values ≤ 0.05 considered significant. Analyses were conducted with Mplus 8.0 and SPSS 27.0.

## Results

### Sample characteristics

The sample had a mean age of 31.67 years (*SD* = 15.71, range 16–78), with the majority (75.6%) identifying as female. Full sample characteristics are shown in Table 1. Of the 1928 participants who provided data, 1926 (99.9%), 738 (38.3%), 527 (27.3%), 513 (26.6%), and 485 (25.2%) responded to surveys at weeks 0, 4, 12, 26 and 52, respectively. Two respondents did not complete the Week 0 survey but provided responses at week 4 and were included in the analyses.

### Identification of suicidal ideation trajectories

The 5-class quadratic model with latent means freely estimated and quadratic slope variance fixed to zero was chosen as the optimal model considering models that converged to proper solutions, model fit, and meaningfulness of classes (see Supplementary File 2).

**Fig. 1:**
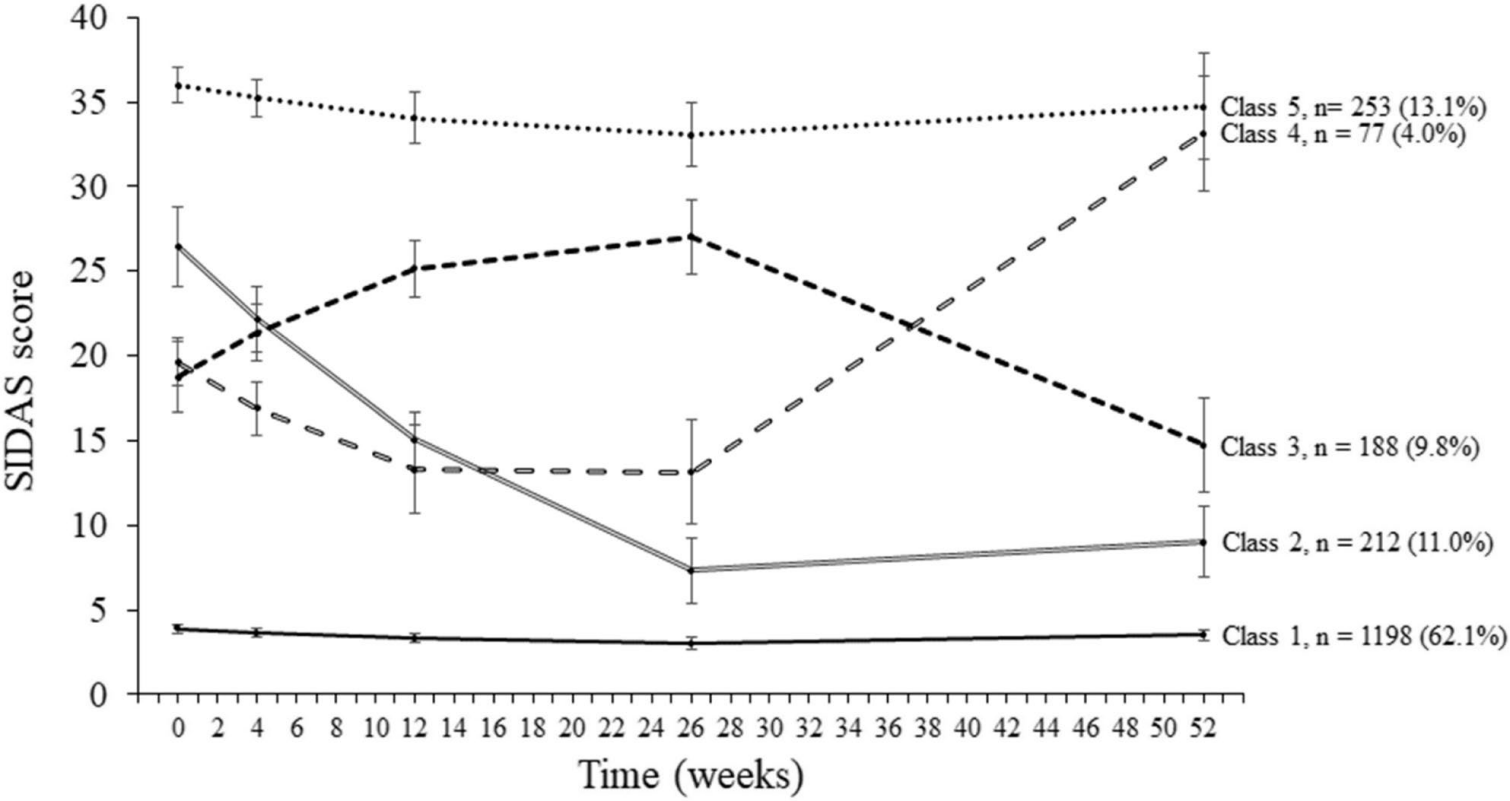
Model estimated means and standard errors for the 5-class model. SIDAS = Suicidal Ideation Attributes Scale

Characteristics of the 5-class model are shown in Table 1 and SIDAS score trajectories are shown in Fig. 1. The class with the lowest level of suicidality (Class 1, *n* = 1198, 62.1% of the sample) was the largest class and had a low stable trajectory of SIDAS scores (all SIDAS scores across timepoints < 4). Class 2 (*n* = 212, 11.0%) had high baseline SIDAS scores (i.e., ≥ 21 threshold indicating high risk of suicidal behaviour) which decreased over time and then stabilised in the low range. Class 3 (*n* = 188, 9.8%) had moderate baseline SIDAS scores which increased into the high range before decreasing back into the moderate range. Class 4 (*n* = 77, 4.0%) had moderate baseline SIDAS scores which decreased before increasing into the high range. The class with the highest level of suicidality (Class 5, *n* = 253, 13.1%) was the second largest class and had a stable trajectory of high SIDAS scores at all timepoints, indicating an elevated risk of suicidal behaviour.

**Table 1:** Baseline descriptive statistics for the whole sample and the 5 classes of the 5-class model

Variable	Full sample (*N* = 1928)	Class 1 (*n* = 1198)	Class 2 (*n* = 212)	Class 3 (*n* = 188)	Class 4 (*n* = 77)	Class 5 (*n* = 253)
Demographics						
Age in years, *M* (*SD*)	31.67 (15.71)	35.15 (16.23)	25.12 (12.43)	27.52 (14.02)	29.45 (14.54)	24.47 (11.68)
Gender identity (female), *n* (%)	1457 (75.6%)	918 (76.6%)	152 (71.7%)	141 (75.0%)	57 (74.0%)	189 (74.7%)
Highest education level (completed post-school qualification), *n* (%)	982 (50.9%)	737 (61.5%)	64 (30.2%)	73 (38.8%)	35 (45.5%)	73 (28.9%)
Relationship status (in a relationship), *n* (%)	878 (45.5%)	612 (51.1%)	75 (35.4%)	84 (44.7%)	20 (26.0%)	87 (34.4%)
Primary outcome						
SIDAS, *M* (*SD*)	12.92 (13.22)	4.16 (5.20)	26.84 (5.51)	18.64 (4.71)	20.32 (5.98)	36.29 (6.23)
Risk variables						
Finance						
Net household income before COVID-19^a^, *Mdn* (range)	4 (1–11)	5 (1–11)	4 (1–11)	4 (1–11)	4 (1–11)	3 (1–11)
Net household income change due to COVID-19^b^, *Mdn* (range)	1 (1–6)	1 (1–6)	1 (1–6)	1 (1–6)	1 (1–6)	1 (1–6)
Economic situation due to COVID-19^c^, *Mdn* (range)	1 (1–4)	1 (1–4)	2 (1–4)	2 (1–4)	2 (1–4)	2 (1–4)
Financial support^d^, *n* (%)	1560 (80.9%)	954 (79.6%)	176 (83.0%)	153 (81.4%)	62 (80.5%)	215 (85.0%)
Housing						
Property ownership^e^, *n* (%)	654 (33.9%)	401 (33.5%)	66 (31.1%)	75 (39.9%)	26 (33.8%)	86 (34.0%)
Housing security due to COVID-19^f^, *Mdn* (range)	1 (1–4)	1 (1–4)	1 (1–4)	1 (1–4)	1 (1–4)	1 (1–4)
Social isolation						
Loneliness^g^, *M* (*SD*)	8.73 (2.35)	8.11 (2.35)	9.86 (2.01)	9.45 (2.02)	9.60 (2.00)	9.92 (1.86)
Social support^h^, *Mdn* (range)	6 (0–6)	6 (0–6)	5 (0–6)	5 (0–6)	5 (0–6)	4 (0–6)
Social support dissatisfaction^i^, *M* (*SD*)	1.92 (0.83)	1.78 (0.77)	2.07 (0.83)	2.12 (0.91)	2.27 (0.85)	2.18 (0.89)
Engagement with community^j^, *Mdn* (range)	1 (0–6)	1 (0–6)	0 (0–5)	0 (0–4)	1 (0–4)	0 (0–4)
EWCEs						
Personally experienced EWCE^k^, *Mdn* (range)	0 (0–4)	0 (0–4)	0 (0–4)	0 (0–4)	0 (0–3)	0 (0–3)
Lived in region affected by EWCE^l^, *Mdn* (range)	1 (1–4)	1 (1–4)	1 (1–4)	1 (1–3)	1 (1–2)	1 (1–3)
Negative effects of any EWCEs^m^, *Mdn* (range)	0 (0–13)	0 (0–13)	0 (0–10)	0 (0–9)	0 (0–13)	0 (0–8)
Employment						
Employment status^n^, *n* (%)	1153 (59.8%)	794 (66.3%)	95 (44.8%)	101 (53.7%)	42 (54.5%)	121 (47.8%)
Employment situation due to COVID-19^o^, *Mdn* (range)	1 (1–4)	1 (1–4)	1 (1–4)	1 (1–4)	1 (1–4)	1 (1–4)
Interpersonal violence and conflict						
Partner interpersonal violence^p^, *M* (*SD*)	6.09 (2.35)	5.91 (1.97)	6.55 (3.01)	6.63 (2.72)	6.28 (2.37)	6.56 (3.49)
Interpersonal conflict/stress in household^q^, *Mdn* (range)	3 (1–5)	2 (1–5)	3 (1–5)	3 (1–5)	3 (1–5)	3 (1–5)
Alcohol and substance use						
Tobacco use^r^, *Mdn* (range)	1 (1–5)	1 (1–5)	1 (1–5)	1 (1–5)	1 (1–5)	1 (1–5)
Alcohol use^r^, *Mdn* (range)	1 (1–5)	1 (1–5)	1 (1–5)	1 (1–5)	2 (1–5)	1 (1–5)
Illicit substance use^r^, *Mdn* (range)	1 (1–5)	1 (1–5)	1 (1–5)	1 (1–5)	1 (1–5)	1 (1–5)
Non-medical prescription medication use^r^, *Mdn* (range)	1 (1–5)	1 (1–5)	1 (1–5)	1 (1–5)	1 (1–5)	1 (1–5)
Mental health						
Lifetime suicide attempt^s^, *n* (%)	734 (38.1%)	313 (26.1%)	115 (54.2%)	98 (52.1%)	33 (42.9%)	175 (69.2%)
Lifetime non-suicidal self-injury^t^, *n* (%)	1236 (64.1%)	611 (51.0%)	179 (84.4%)	155 (82.4%)	63 (81.8%)	228 (90.1%)
PHQ-8^u^, *M* (*SD*)	12.76 (6.22)	10.02 (5.51)	16.84 (4.56)	15.71 (4.06)	15.34 (4.97)	19.33 (3.62)
GAD-7^v^, *M* (*SD*)	10.43 (5.96)	8.25 (5.51)	13.33 (4.89)	12.63 (4.60)	12.43 (4.59)	16.07 (4.34)

Descriptive statistics based on raw (non-imputed) data. *SIDAS* Suicidal Ideation Attributes Scale, *EWCE* Extreme weather and climate event

^a^higher score, higher income. Score of 3 = “$40,001-$60,000”; score of 4 = “$60,001-$80,000”; score of 5 = “$80,001-$100,000”

^b^higher score, greater reduction of income. Score of 1 = “Not changed or increased”

^c^higher score, greater negative impact of COVID-19 on economic situation. Score of 1 = “My economic situation is secure, and will remain secure for the foreseeable future”; score of 2 = “Negative impact due to COVID-19, but is still secure for the foreseeable future”

^d^n (%) refers to those who did not/could not seek financial support from the government

^e^n (%) refers to those who do not own a property

^f^higher score, greater negative impact of COVID-19 on housing security. Score of 1 = “Secure, and unlikely to change in the near future”

^g^higher score, greater loneliness

^h^higher score, greater social support

^i^higher score, greater dissatisfaction with level of social support

^j^higher score, more engagement with community

^k^higher score, greater number of EWCEs personally experienced

^l^higher score, greater number of EWCEs that have directly affected region where participant has lived

^m^higher score, greater number of negative effects of any EWCEs

^n^n (%) refers to those employed

^o^higher score, greater negative impact of COVID-19 on employment situation. Score of 1 = “no change to employment situation”

^p^higher score, more interpersonal violence experienced from partner

^q^higher score, more interpersonal conflict and stress in household. Score of 2 = “ A little”; score of 3 = “Some of the time”

^r^higher score, greater use. Score of 1 = “I didn’t use”; score of 2 = “1-2 times a fortnight”

^s^n (%) refers to those who attempted suicide in their lifetime

^t^n (%) refers to those who engaged in non-suicidal self-injury in their lifetime

^u^higher score, higher depression symptoms

^v^higher score, higher anxiety symptoms

### Associations between baseline risk variables and class membership

For all analyses reported, the class with the lowest level of suicidality (i.e., Class 1) was used as the reference class. Results of preliminary univariate analyses examining baseline risk variables and their associations with classes are shown in Supplementary File 3. Results of multivariate analyses simultaneously examining baseline risk variables within each risk domain and their associations with classes are shown in Table 2 and the summarised in Table 4. Adjusting for other risk variables within a risk domain, we found: (a) seven significant predictors that emerged that were common to all classes (greater negative impacts of COVID-19 on one’s economic situation, greater loneliness, greater number of negative effects of any EWCEs experienced, greater interpersonal conflict and stress in the household, engaging in non-suicidal self-injury in one’s lifetime, and higher depression associated with increased risk of being in Classes 2 to 5; being employed associated with decreased risk of being in Classes 2 to 5), and (b) a unique pattern of additional significant predictors that emerged for each class (see Table 4). In terms of additional significant predictors, notably. Class 4 had only two additional predictors which included a predictor specific to this class (tobacco use), and neither of the two additional predictors were associated with decreased risk of being in this class. Class 2 had six additional predictors, none of which were specific to this class, and two of which were associated with decreased risk of being in this class. Class 3 had eight additional predictors which included two predictors specific to this class (interpersonal violence experienced, not owning a property), and two predictors that were associated with decreased risk of being in this class. Class 5 had ten additional predictors which included two predictors specific to this class (anxiety, social support) and five predictors that were associated with decreased risk of being in this class.

**Table 2:** Baseline risk variables analysed simultaneously within each risk domain by class membership

Variable	Class 2		Class 3		Class 4		Class 5	
	RRR [95% CI]	*p*	RRR [95% CI]	*p*	RRR [95% CI]	*p*	RRR [95% CI]	*p*
Finance								
Net household income before COVID-19^a^	0.89 [0.84, 0.95]	**< 0.001**	0.94 [0.88, 0.99]	0.034	0.98 [0.91, 1.07]	0.672	0.93 [0.88, 0.98]	**0.014**
Net household income change due to COVID-19^b^	1.02 [0.88, 1.17]	0.835	1.01 [0.86, 1.18]	0.920	0.99 [0.79, 1.25]	0.942	0.97 [0.84, 1.12]	0.667
Economic situation due to COVID-19^c^	1.37 [1.11, 1.69]	**0.003**	1.40 [1.12, 1.75]	**0.003**	1.55 [1.13, 2.14]	**0.008**	1.74 [1.43, 2.11]	**< 0.001**
Financial support^d^	1.68 [1.10, 2.55]	0.016	1.48 [0.97, 2.27]	0.070	1.52 [0.80, 2.89]	0.201	2.04 [1.36, 3.06]	**0.001**
Housing								
Property ownership^e^	1.16 [0.77, 1.74]	0.474	1.92 [1.28, 2.88]	**0.003**	0.95 [0.55, 1.63]	0.840	1.50 [0.91, 2.46]	0.113
Housing security due to COVID-19^f^	1.15 [0.96, 1.37]	0.124	1.14 [0.93, 1.41]	0.219	1.07 [0.72, 1.59]	0.725	1.11 [0.90, 1.38]	0.335
Social isolation								
Loneliness^g^	1.39 [1.28, 1.51]	**< 0.001**	1.25 [1.15, 1.35]	**< 0.001**	1.27 [1.12, 1.43]	**< 0.001**	1.34 [1.24, 1.44]	**< 0.001**
Social support^h^	0.95 [0.87, 1.03]	0.175	1.01 [0.92, 1.11]	0.814	0.93 [0.82, 1.05]	0.230	0.86 [0.80, 0.92]	**< 0.001**
Social support dissatisfaction^i^	1.14 [0.93, 1.39]	0.222	1.36 [1.11, 1.66]	**0.003**	1.48 [1.13, 1.94]	**0.005**	1.22 [1.01, 1.47]	**0.038**
Engagement with community^j^	0.89 [0.77, 1.01]	0.077	0.84 [0.72, 0.97]	**0.016**	1.03 [0.85, 1.24]	0.787	0.74 [0.65, 0.86]	**< 0.001**
EWCEs								
Personally experienced EWCE^k^	0.67 [0.52, 0.87]	**0.003**	0.78 [0.60, 1.00]	0.052	0.89 [0.59, 1.35]	0.586	0.74 [0.59, 0.95]	**0.016**
Lived in region affected by EWCE^l^	1.18 [0.81, 1.71]	0.384	1.15 [0.84, 1.56]	0.382	0.90 [0.37, 2.19]	0.807	1.04 [0.76, 1.43]	0.792
Negative effects of any EWCEs^m^	1.33 [1.20, 1.49]	**< 0.001**	1.34 [1.21, 1.50]	**< 0.001**	1.24 [1.05, 1.47]	**0.013**	1.31 [1.18, 1.45]	**< 0.001**
Employment								
Employment status^n^	0.41 [0.31, 0.55]	**< 0.001**	0.59 [0.43, 0.80]	**0.001**	0.60 [0.38, 0.96]	**0.032**	0.46 [0.35, 0.61]	**< 0.001**
Employment situation due to COVID-19^o^	1.14 [0.96, 1.35]	0.134	1.14 [0.91, 1.43]	0.245	1.01 [0.75, 1.36]	0.932	1.07 [0.89, 1.28]	0.458
Interpersonal violence and conflict								
Partner interpersonal violence^p^	1.05 [0.96, 1.14]	0.278	1.11 [1.04, 1.19]	**0.004**	1.03 [0.91, 1.18]	0.641	1.06 [0.99, 1.13]	0.107
Interpersonal conflict/stress in household^q^	1.41 [1.24, 1.61]	**< 0.001**	1.28 [1.12, 1.46]	**< 0.001**	1.29 [1.06, 1.58]	**0.013**	1.74 [1.54, 1.98]	**< 0.001**
Alcohol and substance use								
Tobacco use^r^	1.00 [0.88, 1.14]	0.985	1.01 [0.88, 1.16]	0.866	1.20 [1.01, 1.43]	**0.038**	1.10 [0.98, 1.23]	0.111
Alcohol use^r^	0.86 [0.73, 1.00]	0.053	0.89 [0.76, 1.05]	0.179	0.95 [0.76, 1.20]	0.674	0.93 [0.81, 1.07]	0.335
Illicit substance use^r^	1.49 [1.25, 1.78]	**< 0.001**	1.31 [1.08, 1.60]	**0.007**	0.99 [0.72, 1.37]	0.955	1.27 [1.07, 1.51]	**0.006**
Non-medical prescription medication use^r^	1.39 [1.19, 1.63]	**< 0.001**	1.33 [1.12, 1.58]	**0.001**	1.24 [0.95, 1.62]	0.112	1.58 [1.38, 1.81]	**< 0.001**
Mental health								
Lifetime suicide attempt^s^	2.15 [1.55, 3.00]	**< 0.001**	2.05 [1.46, 2.87]	**< 0.001**	1.45 [0.89, 2.37]	0.112	3.70 [2.59, 5.29]	**< 0.001**
Lifetime non-suicidal self-injury^s^	2.51 [1.65, 3.83]	**< 0.001**	2.37 [1.56, 3.61]	**< 0.001**	2.53 [1.37, 4.67]	**0.003**	3.31 [2.00, 5.48]	**< 0.001**
PHQ-8^t^	1.25 [1.19, 1.31]	**< 0.001**	1.18 [1.13, 1.23]	**< 0.001**	1.16 [1.09, 1.24]	**< 0.001**	1.38 [1.30, 1.45]	**< 0.001**
GAD-7^u^	1.01 [0.97, 1.06]	0.493	1.02 [0.98, 1.06]	0.332	1.02 [0.97, 1.09]	0.433	1.09 [1.04, 1.14]	**< 0.001**

Results in the table are based on the multiple imputation datasets and obtained from separate multivariate multinomial logistic regression analyses conducted for each risk domain to determine the unique predictive variables within each risk domain. All relative risk ratios are therefore pooled statistics based on imputed data. Class 1 with a low stable trajectory of SIDAS scores is the reference class for the relative risk ratios. *RRR* relative risk ratio, *EWCE* Extreme weather and climate event; Bold font = p-value significance level is < 0.05

^a^higher score, higher income

^b^higher score, greater reduction of income

^c^higher score, greater negative impact of COVID-19 on economic situation

^d^0=did not/could not seek financial support from the government, 1=sought financial support from the government (reference group)

^e^0=do not own a property, 1=own a property (reference group)

^f^higher score, greater negative impact of COVID-19 on housing security

^g^higher score, greater loneliness

^h^higher score, greater social support

^i^higher score, greater dissatisfaction with level of social support

^j^higher score, more engagement with community

^k^higher score, greater number of EWCEs personally experienced

^l^higher score, greater number of EWCEs that have directly affected region where participant has lived

^m^higher score, greater number of negative effects of any EWCEs

^n^0=employed, 1=unemployed (reference group)

^o^higher score, greater negative impact of COVID-19 on employment situation

^p^higher score, more interpersonal violence experienced from partner

^q^higher score, more interpersonal conflict and stress in household

^r^ higher score, greater use

^s^0=no (reference group), 1=yes

^t^higher score, higher depression symptoms

^u^higher score, higher anxiety symptoms

The results for the final multivariate analysis simultaneously examining all baseline risk variables across risk domains that were significant in the previous analyses are shown in Table 3 and summarised in Table 4. Adjusting for other risk variables, we found: (a) two significant predictors that emerged that were common to all classes (engaging in non-suicidal self-injury in one’s lifetime and higher depression associated with increased risk of being in Classes 2 to 5), and (b) a unique pattern of additional significant predictors that emerged for each class (see Table 4). In terms of additional significant predictors, Class 4 had only two additional predictors, with neither being specific to this class, and neither associated with decreased risk of being in this class. Class 3 had three additional predictors which included one predictor specific to this class (negative effects of any EWCEs experienced), and none of the additional predictors associated with decreased risk of being in this class. Class 2 had five additional predictors which included three predictors specific to this class (loneliness, illicit substance use, not owning a property) and two predictors associated with decreased risk of being in this class. Class 5 had six additional predictors which included three predictors specific to this class (interpersonal conflict and stress in the household, anxiety, social support) and two predictors associated with decreased risk of being in this class.

**Table 3:** Baseline risk variables across all risk domains analysed simultaneously by class membership

Variable	Class 2		Class 3		Class 4		Class 5	
	RRR [95% CI]	*p*	RRR [95% CI]	*p*	RRR [95% CI]	*p*	RRR [95% CI]	*p*
Finance								
Net household income before COVID-19^a^	0.94 [0.88, 1.01]	0.084	1.01 [0.94, 1.09]	0.753	1.01 [0.92, 1.11]	0.797	0.99 [0.91, 1.07]	0.792
Economic situation due to COVID-19^b^	1.16 [0.95, 1.43]	0.153	1.19 [0.97, 1.45]	0.101	1.34 [1.01, 1.80]	**0.046**	1.36 [1.10, 1.68]	**0.005**
Financial support^c^	1.16 [0.72, 1.87]	0.553	1.17 [0.73, 1.87]	0.524	1.18 [0.60, 2.34]	0.631	1.36 [0.81, 2.30]	0.247
Housing								
Property ownership^d^	0.59 [0.35, 0.98]	**0.049**	1.33 [0.81, 2.18]	0.266	0.59 [0.31, 1.12]	0.112	0.74 [0.39, 1.42]	0.354
Social isolation								
Loneliness^e^	1.13 [1.03, 1.25]	**0.008**	1.03 [0.94, 1.12]	0.594	1.09 [0.95, 1.25]	0.217	0.99 [0.90, 1.10]	0.879
Social support^f^	0.93 [0.85, 1.02]	0.132	1.02 [0.93, 1.13]	0.643	0.92 [0.81, 1.05]	0.222	0.85 [0.77, 0.93]	**0.001**
Social support dissatisfaction^g^	1.00 [0.80, 1.25]	0.969	1.26 [1.01, 1.57]	**0.043**	1.38 [1.03, 1.86]	**0.032**	1.07 [0.86, 1.35]	0.545
Engagement with community^h^	0.98 [0.84, 1.14]	0.765	0.91 [0.78, 1.06]	0.234	1.09 [0.89, 1.33]	0.430	0.89 [0.75, 1.06]	0.197
EWCEs								
Personally experienced EWCE^i^	0.78 [0.59, 1.03]	0.084	0.90 [0.69, 1.17]	0.420	0.94 [0.64, 1.36]	0.732	0.85 [0.64, 1.14]	0.280
Negative effects of any EWCEs^j^	1.08 [0.96, 1.22]	0.209	1.13 [1.01, 1.28]	**0.038**	1.05 [0.88, 1.25]	0.586	0.99 [0.87, 1.13]	0.905
Employment								
Employment status^k^	0.59 [0.42, 0.83]	**0.002**	0.80 [0.56, 1.13]	0.208	0.80 [0.49, 1.30]	0.364	0.69 [0.48, 0.99]	**0.049**
Interpersonal violence and conflict								
Partner interpersonal violence^l^	0.96 [0.88, 1.05]	0.385	1.05 [0.96, 1.14]	0.307	0.95 [0.83, 1.08]	0.425	0.94 [0.86, 1.03]	0.179
Interpersonal conflict/stress in household^m^	1.15 [0.99, 1.34]	0.060	1.08 [0.93, 1.26]	0.299	1.06 [0.85, 1.32]	0.600	1.33 [1.13, 1.55]	**< 0.001**
Alcohol and substance use								
Tobacco use^n^	0.91 [0.78, 1.05]	0.189	0.91 [0.78, 1.05]	0.203	1.15 [0.96, 1.38]	0.142	0.96 [0.83, 1.12]	0.616
Illicit substance use^n^	1.36 [1.11, 1.67]	**0.003**	1.16 [0.94, 1.45]	0.174	0.95 [0.68, 1.34]	0.787	1.12 [0.90, 1.40]	0.321
Non-medical prescription medication use^n^	1.05 [0.88, 1.25]	0.617	1.03 [0.85, 1.24]	0.778	0.97 [0.73, 1.29]	0.855	1.12 [0.94, 1.34]	0.214
Mental health								
Lifetime suicide attempt^o^	2.12 [1.50, 3.02]	**< 0.001**	2.00 [1.41, 2.85]	**< 0.001**	1.55 [0.93, 2.59]	0.096	3.82 [2.61, 5.59]	**< 0.001**
Lifetime non-suicidal self-injury^o^	2.38 [1.54, 3.70]	**< 0.001**	2.20 [1.43, 3.38]	**< 0.001**	2.72 [1.44, 5.15]	**0.002**	3.27 [1.94, 5.53]	**< 0.001**
PHQ-8^p^	1.21 [1.15, 1.27]	**< 0.001**	1.15 [1.10, 1.21]	**< 0.001**	1.13 [1.05, 1.21]	**0.001**	1.34 [1.26, 1.41]	**< 0.001**
GAD-7^q^	1.02 [0.97, 1.06]	0.467	1.02 [0.97, 1.06]	0.471	1.03 [0.96, 1.10]	0.401	1.09 [1.04, 1.14]	**< 0.001**

Results in the table are based on the multiple imputation datasets and obtained from a single final multivariate multinomial logistic regression analysis with 20 predictor variables to determine the unique predictive variables. All relative risk ratios are therefore pooled statistics based on imputed data. Class 1 with a low stable trajectory of SIDAS scores is the reference class for the relative risk ratios. *RRR* relative risk ratio, *EWCEs* Extreme weather and climate events; Bold font = p-value significance level is < 0.05

^a^higher score, higher income

^b^higher score, greater negative impact of COVID-19 on economic situation

^c^0 = did not/could not seek financial support from the government, 1 = sought financial support from the government (reference group)

^d^0 = do not own a property, 1 = own a property (reference group)

^e^higher score, greater loneliness

^f^higher score, greater social support

^g^higher score, greater dissatisfaction with level of social support

^h^higher score, more engagement with community

^i^higher score, greater number of EWCEs personally experienced

^j^higher score, greater number of negative effects of any EWCEs

^k^0 = employed, 1 = unemployed (reference group)

^l^higher score, more interpersonal violence experienced from partner

^m^higher score, more interpersonal conflict and stress in household

^n^higher score, greater use

^o^0 = no (reference group), 1 = yes

^p^higher score, higher depression symptoms

^q^higher score, higher anxiety symptoms

### Associations between cumulative risk index and class membership

The mean cumulative risk index for Classes 1, 2, 3, 4 and 5 were, respectively: -0.14 (*SD* = 0.32), 0.22 (*SD* = 0.32), 0.18 (*SD* = 0.34), 0.14 (*SD* = 0.31) and 0.33 (*SD* = 0.31). The analyses of the cumulative risk index are shown in Table 5. Relative to Class 1, the cumulative risk index was significantly associated with increased risk of being in Classes 2 to 5; and relative to Classes 2 to 4, the cumulative risk index was significantly associated with increased risk of being in Class 5.

Given the previous results, in exploratory analyses, amalgamating Classes 2, 3, and 4 together, relative to Class 1, the cumulative risk index was significantly associated with increased risk of being in the amalgamated classes (RRR = 24.34, 95% CI [16.08, 36.82], *p* < .001) and Class 5 (RRR = 75.68, 95% CI [45.06, 127.14], *p* < .001). Relative to the amalgamated classes, the cumulative risk index was significantly associated with increased risk of being in Class 5 (RRR = 3.11, 95% CI [1.95, 4.97], *p* < .001).

**Table 4:** Pattern of predictors associated with increased or decreased risk of being in a class across the main analyses

	Baseline risk variables analysed simultaneously within each risk domain				Baseline risk variables across all risk domains analysed simultaneously			
	Class 2 predictors	Class 3 predictors	Class 4 predictors	Class 5 predictors	Class 2 predictors	Class 3 predictors	Class 4 predictors	Class 5 predictors
Predictors associated with increased risk across both analyses								
greater negative impacts of COVID-19 on one’s economic situation^a^	↑	↑	↑	↑			↑	↑
greater loneliness^a^	↑	↑	↑	↑	↑			
greater number of negative effects of any EWCEs experienced^a^	↑	↑	↑	↑		↑		
greater interpersonal conflict and stress in the household^a^	↑	↑	↑	↑				↑
engaging in non-suicidal self-injury in one’s lifetime^a, b^	↑	↑	↑	↑	↑	↑	↑	↑
higher depression^a, b^	↑	↑	↑	↑	↑	↑	↑	↑
not seeking or not being able to seek financial support from government	↑			↑				
greater dissatisfaction with the level of social support		↑	↑	↑		↑	↑	
greater partner interpersonal violence experienced		↑						
greater tobacco use			↑					
greater illicit substance use	↑	↑		↑	↑			
greater non-medical prescription medication use	↑	↑		↑				
attempting suicide in one’s lifetime	↑	↑		↑	↑	↑		↑
higher anxiety				↑				↑
Predictors associated with decreased risk across both analyses								
being employed^a^	↓	↓	↓	↓	↓			↓
higher net household income before COVID-19	↓	↓		↓				
greater social support				↓				↓
greater engagement with the community		↓		↓				
greater number of EWCEs personally experienced	↓			↓				
Predictors differentially associated with risk across both analyses								
not owning a property		↑			↓			

For baseline risk variables analysed simultaneously within each risk domain, results in this table summarise results from Table 2. For baseline risk variables across all risk domains analysed simultaneously, results in this table summarise results from Table 3. The class with the lowest level of suicidality (i.e., Class 1) was used as the reference class. ↑ = predictor associated with increased risk of being in class; ↓ = predictor associated with decreased risk of being in class. *EWCE* Extreme weather and climate event

^a^common predictor for all classes when baseline risk variables analysed simultaneously within each risk domain

^b^common predictor for all classes when baseline risk variables across all risk domains analysed simultaneously

**Table 5:** Associations between cumulative risk (CR) index and class membership

Variable	Class 2		Class 3		Class 4		Class 5	
	RRR [95% CI]	*p*	RRR [95% CI]	*p*	RRR [95% CI]	*p*	RRR [95% CI]	*p*
Reference: Class 1								
CR index	30.02 [17.74, 50.79]	**< 0.001**	23.62 [13.85, 40.30]	**< 0.001**	15.04 [6.98, 32.41]	**< 0.001**	76.39 [45.42, 128.47]	**< 0.001**
Reference: Class 2								
CR index	-	-	0.79 [0.43, 1.43]	0.431	0.50 [0.22, 1.17]	0.109	2.55 [1.45, 4.45]	**0.001**
Reference: Class 3								
CR index	-	-	-	-	0.64 [0.27, 1.48]	0.295	3.23 [1.81, 5.78]	**< 0.001**
Reference: Class 4								
CR index	-	-	-	-	-	-	5.08 [2.25, 11.48]	**< 0.001**

Results in the table are based on the multiple imputation datasets and obtained from separate univariate multinomial logistic regression analyses each with a different reference class. All relative risk ratios are therefore pooled statistics based on imputed data; Bold font = p-value significance level is < 0.05

## Discussion

This study explored variation in suicidal ideation trajectories during a period where Australians were facing significant and multiple health (i.e., COVID-19) and environmental (e.g., bushfires, drought) threats. Our results show five different trajectories of suicidal ideation across a one-year period, including stable low (Class 1 – largest class) and stable high (Class 5 – second largest class) trajectories. There were three changing trajectories of suicidal ideation; one that started in the high range and stabilised in the low range (Class 2); and two with moderate suicidal ideation which increased into the high range and stabilised there (Class 4) or returned to the moderate range (Class 3). These findings add to and extend prior research and theory in several ways. First, though our class membership findings align with previous research showing different trends in the prevalence of suicidal ideation during the COVID-19 pandemic (e.g. [26]), , we were also able to demonstrate meaningful heterogeneity in suicidal ideation intensity trajectories. Second, the evidence for both stable and changing trajectory classes may add to current debate as to whether ideation should be treated as a state or trait [41]. That is, even in the “unstable” classes (i.e., classes 2–4) ideation intensity remained at a concerning level that would benefit from psychosocial intervention. This finding suggests that ideation should be considered as a ‘trait’, i.e., a long-term characteristic of an individual that has the potential to affect their behaviour, actions, and feelings. However, the identification of unstable trajectories also shows that the intensity of ideation can change in the moment, for short periods, in response to stressors. In accordance with a “state” argument, classes 2–4 each had at least one point of their suicidal ideation trajectory in the high range (i.e., SIDAS scores ≥ 21) suggesting temporal fluctuation in their vulnerability to enact suicidal thoughts. If ideation is both a trait and a state phenomenon, then individuals identified as having ideation may require access to both long-term (preventative) and just-in-time adaptive intervention approaches to protect against a transition to suicidal behaviour.

Our findings also show that the different ways in which individuals experience health, social, and environmental risk factors contribute to temporal differences in suicidal ideation. Our final adjusted model showed that only two baseline risk variables emerged as consistent predictors for all four vulnerable classes (2 to 5) relative to Class 1. These were a lifetime history of engaging in non-suicidal self-injury, which was associated with 2.20–3.27 times the risk of being in a vulnerable class, and higher depression scores which was associated with 1.13–1.34 times the risk of vulnerability. Outside of these two psychological risk factors, substantial variance in social determinant and mental health risks for class membership was observed, suggesting opportunities for targeted intervention. For example, loneliness, illicit substance use, and suicide attempts appear to be important contributory factors to address for those in Class 2, and social connectedness interventions and addiction treatments may be particularly beneficial for this group. For Class 3, intervening during, and immediately following EWCEs with strong social support programs and policies that aim to minimise distress and mitigate trauma-impacts may help to offset short-term spikes in ideation severity. With negative economic situations and household conflict risks being features of one or both of the most vulnerable classes [4, 5], having strong economic, housing, and welfare policies in place may be a particularly important approach to protecting against suicide attempts. Further research will be needed to test whether addressing the factors specific to each vulnerable class, by reducing those associated with risk or promoting those associated with decreased risk where possible, can ultimately reduce the level of suicidality for these classes.

We also sought to determine whether cumulative risk, i.e., the combined effects of the mental health, social, and environmental risk factors, predicted suicidal ideation trajectories. All vulnerable classes had experienced higher cumulative risk than Class 1, and the most vulnerable class [5] had experienced the highest cumulative risk, showing a strong correlation between risk exposure and suicide vulnerability. This finding is consistent with cumulative risk research in other areas of mental health, which has also shown that individuals exposed to multiple risk factors have worse psychological outcomes than those with less risk factor exposure (e.g. [27, 28]). This finding underscores the utility of a cumulative risk index as a parsimonious approach for operationalising complex, multi-domain risk exposure and distinguishing gradients of vulnerability across suicidal ideation trajectories.

Results from this study have several important implications for research and policy. First, they highlight the heterogeneity of suicidal ideation trajectories and emphasise the importance of research that allows for distinct trajectories to be identified, shaping future research directions. Second, they underscore the importance of viewing suicide risk from a socio-ecological perspective in both longitudinal cohort studies and in government-funded suicide surveillance systems. Incorporating indicators of social determinants, environmental exposures, and health threats in these types of monitoring platforms would help better understand temporal patterns in suicide risk vulnerability, enabling more precise and timely responses. Third, and relatedly, the findings demonstrate the potential utility of a cumulative risk index to (a) help decision-makers make sense of the collective impacts of social-environmental-health exposures on individuals and communities, guiding upstream suicide prevention efforts (e.g., policy changes, funding investment), and (b) be used as a tool to evaluate whether such policies and interventions are effective in reducing vulnerability, and if so, this leads to reductions in population level suicide rates.

The results of this study should be interpreted within the context of several limitations. The study was longitudinal in design and causality between risk factors and suicidal ideation trajectories cannot be inferred. Measures of risk factors in the various risk domains, except those in the mental health domain, were also designed or modified for the purposes of this study, with further evaluation needed in future research. The majority of the sample identified as female, which may affect the generalisability of the results. The SIDAS completion rate was 43.5%, although missing data or participant drop-out is common in other similar research (e.g., 40% participants retained at 1-year follow-up [20]). This study also focused on specific mental health, social, and environmental risk factors at baseline. Unmeasured factors occurring at baseline, as well as how factors evolve after the baseline timepoint, may have been associated with suicidal ideation trajectories. Finally, this study did not model potential complex interactions between the mental health, social, and environmental risk factors, and did not model potential mediational pathways either (e.g., mental health indicators as mediators in the associations between social determinants and specific classes).

In conclusion, this study identified significant variation in longer-term suicidal ideation trajectories, highlighting how unique and cumulative risks cluster to drive these temporal differences. Further examination and understanding of multiple risks in relation to suicide vulnerability will ultimately help to address the burden of suicide.

## Supplementary Information

Supplementary Material 1.

## Data Availability

Due to the sensitive nature of the data in this study it cannot be shared publicly. Please contact the corresponding author for information on approvals required to access the data.
