# Supplementary material for: Exploring the unique and cumulative effects of individual-level and social determinants on suicidal ideation trajectories during health and environmental crises – a longitudinal study of Australians
Source: BMC Public Health. 2026 Mar 2;26:1139. doi: 10.1186/s12889-026-26816-4 (PMC13063781; doi:10.1186/s12889-026-26816-4)
Supplement: Supplementary file 1 — Supplementary Material 1. [file 12889_2026_26816_MOESM1_ESM.docx]

**SUPPLEMENTARY MATERIALS**

**Supplementary File 1 – Risk variables**

There were eight risk domains, each assessed with multiple variables, as follows.

***Finance***

There were four measures of different aspects of financial risk. The first measure reflected participants’ net household income *before* COVID on an 11-point scale (1 = *$0-$20,000* to 11 = *$200,001 or more*). The second measure reflected the net household *income change* due to COVID-19 of participants on a 6-point scale (1 = *Not changed or increased* to 6 = *Reduced by more than 70%*). The third measure reflected participants’ economic situation due to COVID-19 on a 4-point scale (1 = *My economic situation is secure and will remain secure for the foreseeable future* to 4 = *Severe negative impact due to COVID-19 and is in immediate decline*). The fourth measure was binary and reflected participants’ seeking of financial support from the government (0 = *did not/could not seek financial support*, 1 = *sought financial support*).

***Housing***

There were two measures of housing risk. The first measure was binary and reflected whether participants owned a property at the time of the survey (0 = *do not own a property*, 1 = *own a property*). The second measure reflected participants’ housing security due to COVID-19 on a 4-point scale (1 = *Secure, and unlikely to change in the near future* to 4 = *Uncertain due to COVID-19*), with higher scores indicating greater negative impact of COVID-19 on housing security.

***Social isolation***

There were four measures of social isolation risk. The first measure was the UCLA 3-item Loneliness Scale [1] with an additional item (*I have meaningful social contact*; reverse scored) included. Items were rated on a 3-point scale (1 = *Hardly Ever* to 3 = *Often*), with higher sum total scores reflecting greater loneliness. The measure of loneliness had good psychometric properties (Cronbach’s alpha = .78 in the current study). The second measure was an adapted version of the Social Support Questionnaire [2] which had 6 hypothetical situations and produced two measures of social support: (a) whether participants had someone to help them in the situation (Yes/No) and (b) how dissatisfied participants were with the level of help on a 6-point scale (1 = *Very satisfied* to 6 = *Very dissatisfied*). The number of ‘yes’ responses over the 6 situations was taken as a measure of social support and the average of the dissatisfaction ratings was taken as a measure of social support dissatisfaction. The latter measure had good psychometric properties (Cronbach’s alpha = .88 in the current study). The fourth measure listed several community-based activities (e.g., volunteering, attending community organised events) and participants endorsed those they engaged in. Higher scores indicate greater engagement with the community.

***Extreme weather and climate events (EWCEs)***

There were three custom measures of participants’ EWCE-related risk. The first measure listed different EWCEs (e.g., *drought*, *bushfire*, *flood*) and participants endorsed those that they have personally experienced in the past 12 months. Higher scores indicate a greater number of EWCEs personally experienced. The second measure again listed different EWCEs and asked participants to endorse the EWCEs that have directly affected the region in which they lived in the past 12 months. Higher scores indicate a greater number of EWCEs that have directly affected the region where the participant has lived. The third measure listed potential effects of EWCEs (e.g., *My property was damaged/destroyed*, *I became injured*) and participants endorsed those that have happened to them. Higher scores reflect a greater number of negative effects of any EWCEs experienced.

***Employment***

There were two measures of employment-related risk. The first measure was binary and reflected whether participants were employed at the time of the survey (0 = *employed*, 1 = *unemployed*). The second measure reflected participants’ employment situation due to COVID-19 on a 4-point scale (1 = *no change to employment situation* to 4 = *my employment has been terminated due to COVID-19*), with higher scores reflecting greater negative impact of COVID-19 on the participant’s employment situation.

***Interpersonal violence and conflict***

There were two measures of risk related to interpersonal violence and conflict. The first measure was the 5-item Extended Hurt, Insult, Threaten, Scream (E-HITS) Screening Tool [3] which assessed interpersonal violence experienced in the past month. Each item was rated on a 5-point scale (1 = *Never* to 5 = *Frequently*), with higher sum total scores reflecting more interpersonal violence experienced from a partner. The E-HITS has good reliability (e.g., Cronbach’s alpha = .75 in the current study) and validity [3]. The second measure reflected the frequency of conflict, stress, or tension between members of participants’ household on a 5-point scale (1 = *I live alone/it’s very peaceful* to 5 = *All of the time*), with higher scores indicating more interpersonal conflict and stress in the household.

***Alcohol and substance use***

A modified 4-item Tobacco, Alcohol, Prescription medications, and other Substance Self-administered Screen (TAPS-1) [4] was used to produce four measures of risk related to alcohol and substance use. The modified TAPS-1 asked participants about their use of tobacco, alcohol, illicit drugs and non-medical use of prescription medications in the past month. Participants rated frequency of use of each substance on a modified scale (1 = *I didn’t use* to 5 = *Daily*), with higher scores reflecting greater use.

***Mental health***

There were four measures related to mental health. The first measure assessed whether participants had attempted suicide in their lifetime (0 = *no*, 1 = *yes*). The second measure assessed whether participants had engaged in non-suicidal self-injury in their lifetime (0 = *no*, 1 = *yes*). The third measure was the Patient Health Questionnaire – 8-item depression scale (PHQ-8) [5] which assessed depression symptoms in the past two weeks without assessing suicidal or self-injurious thoughts. The PHQ-8 was chosen for this study to prevent overlap with the primary outcome. Each PHQ-8 item was rated on a 4-point scale (0 = *Not at all* to 3 = *Nearly every day*), with higher sum total scores reflecting higher depression levels. The PHQ-8 has good reliability (e.g., Cronbach’s alpha = .89 in the current study) and validity [5]. The fourth measure was the Generalised Anxiety Disorder-7 (GAD-7) [6] which assessed anxiety symptoms in the past two weeks. Each item was rated on a 4-point scale (0 = *Not at all* to 3 = *Nearly every day*), with higher sum total scores reflecting higher anxiety levels. The GAD-7 has good reliability (e.g., Cronbach’s alpha = .91 in the current study) and validity [6].

**References**

1. Hughes ME, Waite LJ, Hawkley LC, Cacioppo JT (2004) A Short Scale for Measuring Loneliness in Large Surveys: Results From Two Population-Based Studies. Res Aging. 26(6):655-72. doi: 10.1177/0164027504268574.

2. Sarason IG, Sarason BR, Shearin EN, Pierce GR (2016) A Brief Measure of Social Support: Practical and Theoretical Implications. Journal of Social and Personal Relationships. 4(4):497-510. doi: 10.1177/0265407587044007.

3. Iverson KM, King MW, Gerber MR, Resick PA, Kimerling R, Street AE, et al. (2015) Accuracy of an intimate partner violence screening tool for female VHA patients: a replication and extension. J Trauma Stress. 28(1):79-82. doi: 10.1002/jts.21985.

4. McNeely J, Wu LT, Subramaniam G, Sharma G, Cathers LA, Svikis D, et al. (2016) Performance of the Tobacco, Alcohol, Prescription Medication, and Other Substance Use (TAPS) Tool for Substance Use Screening in Primary Care Patients. Ann Intern Med. 165(10):690-9. doi: 10.7326/M16-0317.

5. Kroenke K, Strine TW, Spitzer RL, Williams JB, Berry JT, Mokdad AH (2009) The PHQ-8 as a measure of current depression in the general population. J Affect Disord. 114(1-3):163-73. doi: 10.1016/j.jad.2008.06.026.

6. Spitzer RL, Kroenke K, Williams JB, Lowe B (2006) A brief measure for assessing generalized anxiety disorder: the GAD-7. Archives of Internal Medicine. 166(10):1092-7. doi: 10.1001/archinte.166.10.1092.

**Supplementary File 2 – Identification of suicidal ideation trajectories**

A one class quadratic model fit the data better than a one class linear model (see Table S1). Proceeding with a quadratic model, the set of 2-class quadratic models with one or more of latent means, variances, and covariances parameters freely estimated across classes had either convergence issues or a problematic latent variable covariance matrix. An attempt to simplify the simplest 2-class model (i.e., latent means freely estimated) by fixing the quadratic slope variance to zero yielded a proper solution. As such, this simpler model was also tested alongside the other models when examining higher class numbers.

Model testing stopped at the set of 5-class models because the only model that converged to a proper solution had one class with < 5% of the sample. Across models tested, only the 2-, 4-, and 5-class models with latent means freely estimated and quadratic slope variance fixed to zero converged to proper solutions (see Table S1). Considering only these models, the 5-class version of this model had the lowest BIC (notably the BLRT did not distinguish the optimal number of classes), and although there was one class with < 5% of the sample in this model, the trajectory of this class was considered meaningful (i.e., this class had an overall increase in suicidal ideation during the study). Hence, the 5-class model was selected as the optimal solution.

For this 5-class model, overall class separation was good (entropy = .77), although there appeared to be some uncertainty in class assignment for Classes 2 and 3 (average posterior probabilities = .95 [Class 1], .59 [Class 2], .55 [Class 3], .71 [Class 4], .83 [Class 5]).

Table S1. Model fit indices for growth mixture models tested

| Model | # free parameters | BIC | BLRT |
| --- | --- | --- | --- |
| 1 class models |  |  |  |
| 1 class linear | 10 | 31167.81 | - |
| 1 class quadratic | 14 | 31154.17 | - |
| 2 class models |  |  |  |
| 2 class quadratic – latent M free | 18 | 30631.18^a^ | - |
| 2 class quadratic – latent M, V free |  | -^b^ | - |
| 2 class quadratic – latent M, V, C free |  | -^b^ | - |
| 2 class quadratic – latent M free; quadratic slope variance fixed to zero | 15 | 30634.37 | *p* < .001 |
| 3 class models |  |  |  |
| 3 class quadratic – latent M free | 22 | 30419.14^a^ | - |
| 3 class quadratic – latent M, V free |  | -^b^ | - |
| 3 class quadratic – latent M, V, C free |  | -^b^ | - |
| 3 class quadratic – latent M free; quadratic slope variance fixed to zero | 19 | 30415.67^a^ | *p* < .001 |
| 4 class models |  |  |  |
| 4 class quadratic – latent M free | 26 | 30294.81^a^ | - |
| 4 class quadratic – latent M, V free |  | -^b^ | - |
| 4 class quadratic – latent M, V, C free |  | -^b^ | - |
| 4 class quadratic – latent M free; quadratic slope variance fixed to zero | 23 | 30278.39 | *p* < .001 |
| 5 class models |  |  |  |
| 5 class quadratic – latent M free | 30 | 30203.97^a^ | - |
| 5 class quadratic – latent M, V free |  | -^b^ | - |
| 5 class quadratic – latent M, V, C free |  | -^b^ | - |
| 5 class quadratic – latent M free; quadratic slope variance fixed to zero | 27 | 30184.13 | *p* < .001 |

*Note*. M = means; V = variances; C = covariances; BIC = Bayesian Information Criterion; BLRT = Bootstrap Likelihood Ratio Test.

^a^ latent variable covariance matrix not positive definite

^b^ did not converge

**Supplementary File 3 – Univariate analyses examining baseline risk variables and their associations with classes**

Table S2 shows the baseline risk variables and their association with each of the classes relative to the class with the lowest level of suicidality (i.e., Class 1). Common predictors for all classes were as follows. Greater negative impacts of COVID-19 on one’s economic situation, greater loneliness, greater dissatisfaction with the level of social support, greater number of negative effects of any EWCEs experienced, greater interpersonal conflict and stress in the household, attempting suicide in one’s lifetime, engaging in non-suicidal self-injury in one’s lifetime, higher depression, and higher anxiety were all significantly associated with increased risk of being in Classes 2 to 5 (RRR range = 1.15-9.13), whereas greater social support and being employed were all significantly associated with decreased risk of being in Classes 2 to 5 (RRR range = 0.41-0.88).

There were additional predictors that were not common to all classes. Greater partner interpersonal violence experienced, greater illicit substance use, and greater non-medical prescription medication use were significantly associated with increased risk of being in Classes 2, 3, and 5 (RRR range = 1.08-1.64), whereas higher net household income before COVID-19 and greater engagement with the community were significantly associated with decreased risk of being in Classes 2, 3 and 5 (RRR range = 0.66-0.97). Greater tobacco use was significantly associated with increased risk of being in Classes 2, 4, and 5 (RRR range = 1.13-1.20). Not owning a property was significantly associated with increased risk of being in Class 3 (RRR = 2.00), and not seeking, or not being able to seek, financial support from the government was significantly associated with increased risk of being in Class 5 (RRR = 1.51).

Table S2. Baseline risk variables analysed individually: Associations between baseline risk variables and class membership

|  | Class 2 |  | Class 3 |  | Class 4 |  | Class 5 |  |
| --- | --- | --- | --- | --- | --- | --- | --- | --- |
| Variable | RRR [95% CI] | *p* | RRR [95% CI] | *p* | RRR [95% CI] | *p* | RRR [95% CI] | *p* |
| **Finance** |  |  |  |  |  |  |  |  |
| Net household income before COVID-19^a^ | 0.89 [0.84, 0.94] | < .001 | 0.93 [0.87, 0.98] | .012 | 0.97 [0.89, 1.05] | .407 | 0.91 [0.86, 0.96] | .002 |
| Net household income change due to COVID-19^b^ | 1.08 [0.96, 1.21] | .197 | 1.09 [0.96, 1.24] | .172 | 1.12 [0.93, 1.34] | .427 | 1.11 [1.00, 1.24] | .058 |
| Economic situation due to COVID-19^c^ | 1.40 [1.18, 1.65] | < .001 | 1.41 [1.18, 1.68] | < .001 | 1.49 [1.16, 1.93] | .002 | 1.65 [1.42, 1.92] | < .001 |
| Financial support^d^ | 1.33 [0.89, 2.00] | .166 | 1.20 [0.80, 1.80] | .389 | 1.21 [0.66, 2.24] | .543 | 1.51 [1.03, 2.22] | .035 |
| **Housing** |  |  |  |  |  |  |  |  |
| Property ownership^e^ | 1.21 [0.81, 1.81] | .350 | 2.00 [1.37, 2.93] | .001 | 0.97 [0.58, 1.62] | .905 | 1.55 [0.97, 2.49] | .075 |
| Housing security due to COVID-19^f^ | 1.16 [0.98, 1.38] | .064 | 1.21 [0.99, 1.46] | .064 | 1.07 [0.73, 1.56] | .732 | 1.15 [0.94, 1.41] | .187 |
| **Social isolation** |  |  |  |  |  |  |  |  |
| Loneliness^g^ | 1.44 [1.34, 1.56] | < .001 | 1.30 [1.21, 1.40] | < .001 | 1.35 [1.20, 1.51] | < .001 | 1.47 [1.37, 1.58] | < .001 |
| Social support^h^ | 0.82 [0.76, 0.88] | < .001 | 0.88 [0.81, 0.95] | .001 | 0.80 [0.72, 0.89] | < .001 | 0.74 [0.69, 0.79] | < .001 |
| Social support dissatisfaction^i^ | 1.54 [1.28, 1.84] | < .001 | 1.64 [1.36, 1.97] | < .001 | 1.89 [1.48, 2.41] | < .001 | 1.73 [1.46, 2.04] | < .001 |
| Engagement with community^j^ | 0.80 [0.70, 0.91] | .001 | 0.78 [0.67, 0.90] | .001 | 0.95 [0.78, 1.14] | .560 | 0.66 [0.57, 0.76] | < .001 |
| **EWCEs** |  |  |  |  |  |  |  |  |
| Personally experienced EWCE^k^ | 0.93 [0.75, 1.15] | .506 | 1.09 [0.89, 1.33] | .407 | 1.07 [0.78, 1.45] | .685 | 0.98 [0.81, 1.19] | .855 |
| Lived in region affected by EWCE^l^ | 1.13 [0.79, 1.61] | .488 | 1.14 [0.85, 1.52] | .388 | 0.92 [0.41, 2.05] | .826 | 1.02 [0.76, 1.37] | .899 |
| Negative effects of any EWCEs^m^ | 1.22 [1.11, 1.33] | < .001 | 1.26 [1.15, 1.39] | < .001 | 1.19 [1.03, 1.37] | .019 | 1.21 [1.11, 1.32] | < .001 |
| **Employment** |  |  |  |  |  |  |  |  |
| Employment status^n^ | 0.41 [0.30, 0.55] | < .001 | 0.58 [0.43, 0.79] | .001 | 0.60 [0.38, 0.96] | .032 | 0.46 [0.35, 0.61] | < .001 |
| Employment situation due to COVID-19^o^ | 1.16 [0.97, 1.38] | .103 | 1.15 [0.91, 1.45] | .232 | 1.02 [0.75, 1.37] | .921 | 1.08 [0.90, 1.30] | .412 |
| **Interpersonal** **violence and conflict** |  |  |  |  |  |  |  |  |
| Partner interpersonal violence^p^ | 1.11 [1.02, 1.20] | .016 | 1.16 [1.08, 1.24] | < .001 | 1.08 [0.95, 1.22] | .243 | 1.15 [1.08, 1.22] | < .001 |
| Interpersonal conflict/stress in household^q^ | 1.45 [1.28, 1.63] | < .001 | 1.37 [1.20, 1.55] | < .001 | 1.32 [1.09, 1.59] | .004 | 1.80 [1.60, 2.03] | < .001 |
| **Alcohol and substance use** |  |  |  |  |  |  |  |  |
| Tobacco use^r^ | 1.13 [1.02, 1.26] | .025 | 1.09 [0.97, 1.22] | .153 | 1.20 [1.03, 1.41] | .019 | 1.20 [1.10, 1.32] | < .001 |
| Alcohol use^r^ | 0.96 [0.83, 1.12] | .621 | 0.97 [0.84, 1.14] | .741 | 1.04 [0.84, 1.30] | .702 | 1.08 [0.95, 1.23] | .243 |
| Illicit substance use^r^ | 1.52 [1.31, 1.77] | < .001 | 1.34 [1.13, 1.60] | .001 | 1.15 [0.85, 1.54] | .372 | 1.45 [1.25, 1.68] | < .001 |
| Non-medical prescription medication use^r^ | 1.45 [1.25, 1.69] | < .001 | 1.36 [1.15, 1.60] | < .001 | 1.27 [0.98, 1.64] | .069 | 1.64 [1.44, 1.88] | < .001 |
| **Mental health** |  |  |  |  |  |  |  |  |
| Lifetime suicide attempt^s^ | 3.35 [2.48, 4.51] | < .001 | 3.07 [2.25, 4.21] | < .001 | 2.17 [1.36, 3.48] | .001 | 6.43 [4.77, 8.65] | < .001 |
| Lifetime non-suicidal self-injury^s^ | 5.19 [3.52, 7.66] | < .001 | 4.50 [3.04, 6.66] | < .001 | 4.31 [2.39, 7.77] | < .001 | 9.13 [5.91, 14.12] | < .001 |
| PHQ-8^t^ | 1.29 [1.24, 1.33] | < .001 | 1.22 [1.18, 1.26] | < .001 | 1.21 [1.15, 1.26] | < .001 | 1.49 [1.42, 1.55] | < .001 |
| GAD-7^u^ | 1.19 [1.15, 1.22] | < .001 | 1.16 [1.12, 1.19] | < .001 | 1.15 [1.10, 1.20] | < .001 | 1.34 [1.29, 1.38] | < .001 |

*Note.* Results in the table are based on the multiple imputation datasets and obtained from separate univariate multinomial logistic regression analyses each examining a single baseline risk variable to determine that variable’s associations with class. All relative risk ratios are therefore pooled statistics based on imputed data. Class 1 with a low stable trajectory of SIDAS scores is the reference class for the relative risk ratios. RRR = relative risk ratio; EWCE = Extreme weather and climate event

^a^ higher score, higher income

^b^ higher score, greater reduction of income

^c^ higher score, greater negative impact of COVID-19 on economic situation

^d^ 0=did not/could not seek financial support from the government, 1=sought financial support from the government (reference group)

^e^ 0=do not own a property, 1=own a property (reference group)

^f^ higher score, greater negative impact of COVID-19 on housing security

^g^ higher score, greater loneliness

^h^ higher score, greater social support

^i^ higher score, greater dissatisfaction with level of social support

^j^ higher score, more engagement with community

^k^ higher score, greater number of EWCEs personally experienced

^l^ higher score, greater number of EWCEs that have directly affected region where participant has lived

^m^ higher score, greater number of negative effects of any EWCEs

^n^ 0=employed, 1=unemployed (reference group)

^o^ higher score, greater negative impact of COVID-19 on employment situation

^p^ higher score, more interpersonal violence experienced from partner

^q^ higher score, more interpersonal conflict and stress in household

^r^ higher score, greater use

^s^ 0=no (reference group), 1=yes

^t^ higher score, higher depression symptoms

^u^ higher score, higher anxiety symptoms

**Supplementary File 4 – Study Survey**

**Demographics**

| **What the participant will see** | | **Drop down response options available** |
| --- | --- | --- |
| Age | --- select ---- | Ages 16 to 100 will be shown in a drop down menu |
| Gender identity | --- select ---- | Female  Male  Non-binary |
| Sex assigned at birth | --- select ---- | Female  Male |
| What State do you live in? | -- select ---- | NSW  QLD  VIC  TAS  SA  WA  NT  ACT |
| What is your suburb of residence? |  |  |
| What is your postcode? |  |  |
| Which of the following best describes your area | -- select ---- | Remote/Rural  Urban/Metropolitan |
| Do you identify as being Lesbian, Gay, Bisexual, Transgender, Intersex, Queer, or other? | -- select ---- | No  Lesbian  Gay  Bisexual  Transgender  Intersex  Queer  Other  Prefer not to say |
| Indigenous status | -- select ---- | Not Aboriginal or Torres Strait Islander  Yes – Aboriginal  Yes, Torres Strait Islander Yes – both  I prefer not to say |
| Language/s spoken at home | -- select ---- | English only  English and other language  Other language only |
| Current living situation | -- select ---- | Live alone  Live with parent/s/family,  Live with a significant other  Live with roommate(s)  Other |
| Current relationship situation | -- select ---- | Not in a relationship  Partnered/dating  De facto/married  Separated  Divorced or widowed  Other |
| Highest level of education completed | -- select ---- | Primary school  Years 7 to 9  Year 10 or equivalent  Year 11  Year 12 or equivalent  Certificate Level I - IV Diploma/Associate Degree  Graduate Diploma/Certificate  Bachelor Degree  Master degree  Doctoral degree |
| What is your current employment status? | -- select ---- | Full-time  Part-time  Casual  Self-employed  Unemployed |
| What is your usual main profession? |  | [free text typing option] |

**Mental health questions**

| **Patient Health Questionnaire-9 (PHQ-9)^2^** | | | | |
| --- | --- | --- | --- | --- |
| **Over the past 2 weeks, how often have you been bothered by the following problems?** | **Not at all** | **Several days** | **More than half the days** | **Nearly every day** |
| Little interest or pleasure in doing things | 0 | 1 | 2 | 3 |
| Feeling down, depressed, irritable, or hopeless | 0 | 1 | 2 | 3 |
| Trouble falling asleep, staying asleep, or sleeping too much | 0 | 1 | 2 | 3 |
| Feeling tired, or having little energy | 0 | 1 | 2 | 3 |
| Poor appetite, weight loss, or overeating | 0 | 1 | 2 | 3 |
| Feeling bad about yourself — or feeling that you are a failure, or that you have let yourself or your family down | 0 | 1 | 2 | 3 |
| Trouble concentrating on things, such as reading the  newspaper or watching television | 0 | 1 | 2 | 3 |
| Moving or speaking so slowly that other people could have noticed? Or the opposite — being so fidgety or restless that you were moving around a lot more than usual | 0 | 1 | 2 | 3 |
| Thoughts that you would be better off dead, or of  hurting yourself in some way | 0 | 1 | 2 | 3 |

| **Generalised Anxiety Disorder-7 (GAD-7)^3^** | | | | |
| --- | --- | --- | --- | --- |
| **Over the past 2 weeks, how often have you been bothered by the following problems?** | **Not at all** | **Several days** | **More than half the days** | **Nearly every day** |
| Feeling nervous, anxious, or on edge | 0 | 1 | 2 | 3 |
| Not being able to stop or control worrying | 0 | 1 | 2 | 3 |
| Worrying too much about different things | 0 | 1 | 2 | 3 |
| Trouble relaxing | 0 | 1 | 2 | 3 |
| Being so restless that it’s hard to sit still | 0 | 1 | 2 | 3 |
| Becoming easily annoyed or irritable | 0 | 1 | 2 | 3 |
| Feeling afraid as if something awful might happen | 0 | 1 | 2 | 3 |

| **Suicidal Ideation Attributes Scale (SIDAS)^1^**  **Now we’re going to ask you some questions about suicidal thoughts, and the severity of them. Please think about the past month when answering the following questions.** | | | |
| --- | --- | --- | --- |
| Question | Response options | | |
| In the past month, how often have you had thoughts about suicide? | 0=Never | 1,2,3,4,5,6,7,8,9 | 10=Always |
| In the past month, how much control have you had over these thoughts? (reversed scoring) | 0=No control | 1,2,3,4,5,6,7,8,9 | 10=Full control |
| In the past month, how close have you come to making a suicide attempt? | 0=Not close at all | 1,2,3,4,5,6,7,8,9 | 10= made an attempt |
| In the past month, to what extent have you felt tormented by thoughts about suicide? | 0=Not at all | 1,2,3,4,5,6,7,8,9 | 10=Extremely |
| In the past month, how much have thoughts about suicide interfered with your ability to carry out daily activities, such as  work, household tasks or social activities? | 0=Not at all | 1,2,3,4,5,6,7,8,9 | 10=Extremely |

| **Intentional self-harm questions (created for this study)**  **The following questions ask whether you’ve ever harmed yourself on purpose, or attempted to take your own life.** | |
| --- | --- |
| Question | Response options |
| Have you ever attempted suicide? | No, never  Yes, once  Yes, more than once |
| If ‘Yes, more than once’ then:  How many times have you attempted suicide? | 2, 3, 4, 5, 6+ |
| When was your most recent suicide attempt? | More than 12months ago  Within the last 12 months  Within the last month |
| Have you ever injured yourself on purpose (i.e., intentional, self-inflicted damage to the surface of the body without suicidal intent) | No  Yes |
| When was your most recent self-injury? | More than 12months ago  Within the last 12 months  Within the last month |
| If ‘within the last month’ then:  In the past month, how often have you injured yourself on purpose? | Once  2-5 times  6-9 times  10-20 times  20+ times |
| Please rate how serious your worst injury was in the past month | No care was needed  Some care was needed  I required medical care |

| **Distress Questionnaire-5 (DQ-5)^4^** | | | | | |
| --- | --- | --- | --- | --- | --- |
| **Thinking about the past month, how often were you bothered by the following problems?** | **Never** | **Rarely** | **Sometimes** | **Often** | **Always** |
| My worries overwhelmed me | 1 | 2 | 3 | 4 | 5 |
| I felt hopeless | 1 | 2 | 3 | 4 | 5 |
| I found social settings upsetting | 1 | 2 | 3 | 4 | 5 |
| I had trouble staying focused on tasks | 1 | 2 | 3 | 4 | 5 |
| Anxiety or fear interfered with my ability to do the things I needed to do at work or at home | 1 | 2 | 3 | 4 | 5 |

| **Short Grit Scale (SGS)^5,6^** | | | | | |
| --- | --- | --- | --- | --- | --- |
| Here are a number of statements that may or may not apply to you. When responding, think of how you compare to most people - not just the people you know well, but most people in the world. There are no right or wrong answers, so just answer as honestly as you can. | **Not like me at all** | **Not much like me** | **Somewhat like me** | **Mostly like me** | **Very much like me** |
| New ideas and projects sometimes distract me from previous ones | 5 | 4 | 3 | 2 | 1 |
| Setbacks don’t discourage me | 1 | 2 | 3 | 4 | 5 |
| I have been obsessed with a certain idea or project for a short time but later lost interest. | 5 | 4 | 3 | 2 | 1 |
| I am a hard worker | 1 | 2 | 3 | 4 | 5 |
| I often set a goal but later choose to pursue a different one | 5 | 4 | 3 | 2 | 1 |
| I have difficulty maintaining my focus on projects that take more than a few months to complete | 5 | 4 | 3 | 2 | 1 |
| I finish whatever I begin | 1 | 2 | 3 | 4 | 5 |
| I am diligent | 1 | 2 | 3 | 4 | 5 |

| **The Tobacco, Alcohol, Prescription medications, and other Substance (TAPS-1) Self-administered Screen (modified for this study)^7^** | | | | | |
| --- | --- | --- | --- | --- | --- |
| **On average, in the past month, how often have you:** | **I didn’t use** | **1-2 times a fortnight** | **1-3 times a week** | **More than 4 times a week** | **Daily** |
| Used any tobacco product (for example, cigarettes, e- cigarettes, cigars, pipes, or smokeless tobacco) | 1 | 2 | 3 | 4 | 5 |
| Had 4 or more drinks containing alcohol in one day (one standard drink is 1x small glass of wine, 1x beer, or 1x single shot of liquor) | 1 | 2 | 3 | 4 | 5 |
| Used any illicit drugs including marijuana, cocaine or crack, heroin, methamphetamine (crystal meth), hallucinogens, ecstasy/MDMA | 1 | 2 | 3 | 4 | 5 |
| Used any prescription medications just for the feeling, more than prescribed or that were not prescribed for you (e.g., opiate pain relievers, medications for anxiety or sleeping, or for ADHD) | 1 | 2 | 3 | 4 | 5 |

| **Actual Help-Seeking Questionnaire (AHSQ)^8,9^** |
| --- |
| **Below is a list of people who you might seek help or advice from if you were experiencing a personal or emotional problem. Please select any of these who you have gone to for advice or help in the past 2 weeks for a personal or emotional problem. Check all that apply:** |
| Partner (e.g., significant boyfriend or girlfriend) |
| Friend (not related to you) |
| Parent |
| Other relative / family member |
| Mental health professional (e.g., counsellor, psychologist, psychiatrist) |
| Online help services (e.g., Beyond Blue web chat) |
| Phone help line (e.g., Lifeline) |
| Family doctor/ GP |
| I have not sought help from anyone for my problem |

|  | **Help receipt (designed for this survey)** |  |
| --- | --- | --- |
| 1 | Have you ever experienced mental illness or been diagnosed with mental illness? | Yes  No  I don’t know |
| 2 | Have you ever seen a mental health professional (counsellor, psychologist, psychiatrist) for a mental health problem? | Yes  No  I don’t know |
| 3 | Are you **currently** receiving psychological help for a mental health problem? | Yes  No  I don’t know. |
| 4 | If yes to Q3: How many months have you been receiving this help for? (please answer to the nearest month) | 1, 2, 3, 4, 5, 6, 7, 8, 9, 10, 11, 12, 18, 24, 36, 48+ |

|  | **Pandemic (designed for this survey) We are now going to ask you some questions in relation to the coronavirus (COVID-19) pandemic.** | |
| --- | --- | --- |
|  | **Question** | **Response Options** |
| 1 | Please describe the current state of your health in relation to COVID-19. Please check all that apply to you. | I have been diagnosed, and currently have COVID-19  I have been diagnosed and have recovered from COVID-19  I am suspected to have COVID-19, or am waiting test results  I have no symptoms of COVID-19  I have no symptoms, but someone in my household has recently been diagnosed with COVID-19 |
| 2 | Please describe your current employment situation. Check all that apply: | Student  Employer/ business owner  Employee  Unemployed |
| 3 | If ‘ employee’ or ‘unemployed’ then: Please describe if your current employment situation has changed as a result of COVID-19. Please check all that apply to you. | No change  My employment status has been modified (e.g. reduced hours)  My employment has been terminated until further notice (I.e. stood down)  My employment has been terminated due to COVID-19 |
| 3.1 | If there has been a change in the employment situation then: How long has this change been in effect? | Less than one month  1-2 months  2-4 months  4+ months |
| 4 | If ‘employer/ business owner’ then:  To what degree has your business been negatively impacted by COVID-19? | None, business has improved  None, it’s business as usual  A little  A lot  Business has closed I have lost my business |
| 5 | If ‘student’ or ‘employee’ in Q2 then: Has your school / university / workplace shut down? | Yes  No |
| 5.1 | If yes to Q5 then: How long has this been in effect? | Less than one month  1-2 months  2-4 months  4+ months |
| 6 | Have you sought or any government financial relief packages? | No  Yes, JobKeeper Payment (as an employer)  Yes, JobKeeper Payment (as an employee)  Yes, boosting cash flow for employers  Yes, early release of superannuation |

|  | **Psychosocial safety climate, presenteeism/absenteeism, satisfaction, work-family integration blurring scale (Created for this survey)** |  |
| --- | --- | --- |
| If selected ‘employee’ in Q2 above:  In my organisation/workplace: | | |
| 1.1 | Senior management show support for stress prevention through involvement and commitment | 1. Strongly disagree  2. 3. 4.  5. Strongly agree |
| 1.2 | Senior management considers employee psychological health to be as important as productivity | 1. Strongly disagree  2. 3. 4.  5. Strongly agree |
| 1.3 | There is good communication here about psychological safety issues which affect me | 1. Strongly disagree  2. 3. 4.  5. Strongly agree |
| 1.4 | The prevention of stress involves all levels of the organisation | 1. Strongly disagree  2. 3. 4.  5. Strongly agree |
| 2.1 | To what extent has COVID-induced stress/problems affected your performance in your paid work? | 1. Not at all  2. 3. 4. 5. 6. 7. 8. 9.  10. Worst you can imagine |
| 2.2 | How difficult have COVID-induced problems with your health (mental or physical) made it for you to do your paid work to your best ability? | 1. Not at all  2. 3. 4. 5. 6. 7. 8. 9.  10. Worst you can imagine |
| 2.3 | How frequently did COVID-induced stress/problems make you unable to do any of your paid work (i.e., need to take days off)? | 1. Not at all  2. 3. 4. 5. 6. 7. 8. 9.  10. Worst you can imagine |
| 3 | Taking everything into consideration, how do you feel about your job as a whole? | 1. Extremely dissatisfied  2. 3. 4. 5. 6. 7. 8. 9.  10. Extremely satisfied |
| 4.1 | If you have been working from home during the Covid period:  I’ve been tending to integrate my paid-work duties with my personal life | 0. Haven’t worked from home  1. Strongly disagree  2. 3. 4.  5. Strongly agree |
| 4.2 | There has been a somewhat blurred boundary between my paid-work roles and my other personal roles | 0. Haven’t worked from home  1. Strongly disagree  2. 3. 4.  5. Strongly agree |
| 4.3 | It has often been difficult to tell where my paid-work life ends and my personal life begins | 0. Haven’t worked from home  1. Strongly disagree  2. 3. 4.  5. Strongly agree |
| 4.4 | As a result of working from home, it has become more difficult to separate my paid-work duties from my personal life | 0. Haven’t worked from home  1. Strongly disagree  2. 3. 4.  5. Strongly agree |
| If selected ‘student’ in Q2 Pandemic survey above:  In my school/college/university: | | |
| 5.1 | The staff seem to be involved and committed in preventing/reducing student stress | 1. Strongly disagree  2. 3. 4.  5. Strongly agree |
| 5.2 | The staff consider student psychological health/wellbeing to be as important as productivity | 1. Strongly disagree  2. 3. 4.  5. Strongly agree |
| 5.3 | There is good communication about psychological/wellbeing issues which affect me | 1. Strongly disagree  2. 3. 4.  5. Strongly agree |
| 5.4 | The prevention of student stress involves all levels of the educational institute (i.e., instructors, students, and senior management) | 1. Strongly disagree  2. 3. 4.  5. Strongly agree |
| 6.1 | To what extent has COVID-induced stress/problems affected your performance in your studies? | 1. Not at all  2. 3. 4. 5. 6. 7. 8. 9.  10. Worst you can imagine |
| 6.2 | How difficult have COVID-induced problems with your health (mental or physical) made it for you to do your university work to your best ability? | 1. Not at all  2. 3. 4. 5. 6. 7. 8. 9.  10. Worst you can imagine |
| 6.3 | How frequently did COVID-induced stress/problems make you unable to do any of your university work (i.e., need to take days off)? | 1. Not at all  2. 3. 4. 5. 6. 7. 8. 9.  10. Worst you can imagine |
| 7 | Taking everything into consideration, how do you feel about your studies as a whole? | 1. Extremely dissatisfied  2. 3. 4. 5. 6. 7. 8. 9.  10. Extremely satisfied |

|  | **Social Support (Adapted from a brief measure of social support)^10^** | |
| --- | --- | --- |
|  | **The following questions ask about people in your life who provide you with help or support. In the first question, you will be asked if you have someone in your life which meets the description. For the second part, please indicate how satisfied you are with the overall support you have.** | **Responses** |
| 1 | I have someone I can count on when I need help | Yes  No |
| 1.1 | How satisfied are you with this level of support? | Very satisfied  Fairly satisfied  A little satisfied  A little dissatisfied  Fairly dissatisfied  Very dissatisfied |
| 2 | I have someone I can really count on to feel more relaxed when I am under pressure or tense | Yes  No |
| 2.1 | How satisfied are you with this level of support? | Very satisfied  Fairly satisfied  A little satisfied  A little dissatisfied  Fairly dissatisfied  Very dissatisfied |
| 3 | I have someone who accepts me totally, including both my worst and best traits | Yes  No |
| 3.1 | How satisfied are you with this level of acceptance? | Very satisfied  Fairly satisfied  A little satisfied  A little dissatisfied  Fairly dissatisfied  Very dissatisfied |
| 4 | I have someone I can count on to care about me, regardless of what is happening to me | Yes  No |
| 4.1 | How satisfied are you with this level of care? | Very satisfied  Fairly satisfied  A little satisfied  A little dissatisfied  Fairly dissatisfied  Very dissatisfied |
| 5 | I have someone I can really count on to help me feel better when I am feeling low | Yes  No |
| 5.1 | How satisfied are you with this level of support? | Very satisfied  Fairly satisfied  A little satisfied  A little dissatisfied  Fairly dissatisfied  Very dissatisfied |
| 6 | I have someone I can count on to console me when I am very upset | Yes  No |
| 6.1 | How satisfied are you with this level of support? | Very satisfied  Fairly satisfied  A little satisfied  A little dissatisfied  Fairly dissatisfied  Very dissatisfied |

| **COVID-19 exacerbation and worry (created for the LifeBuoy trial)**  **Please answer the following questions about your mental health in relation to the coronavirus (COVID-19) pandemic.** | | |
| --- | --- | --- |
| Questions | Response options | |
| Do you think the COVID-19 pandemic has increased your anxiety levels more than usual? | -- select one -- | 0=I haven’t had feelings of anxiety  1=not at all  2=a little  3=some of the time  4= a lot  5=all of the time |
| Do you think the COVID-19 pandemic has increased your depression levels more than usual? | -- select one -- | 0=I haven’t had feelings of depression  1=not at all  2=a little  3=some of the time  4= a lot  5=all of the time |
| If you have had recent suicidal thoughts, do you think the COVID-19 pandemic has increased the frequency or severity of these thoughts? | -- select one -- | 0=I haven’t had recent suicidal thoughts  1=not at all  2=a little  3=some of the time  4= a lot  5=all of the time |
| If you were to develop flu-like symptoms tomorrow, would you be worried? | -- select one -- | 1=not at all worried  2=much less worried than normal  3=worried less than normal  4=about the same  5=worried more than normal  6=worried much more than normal  7=extremely worried |
| In the past one week, have you ever worried about catching COVID-19? | -- select one -- | 1=no, never think about it  2=think about it but it didn't worry me  3=worried me a bit  4=worried me a lot  5=worried about it all the time |
| Please rate the current level of your worry towards COVID-19 | -- select one -- | 1 =very mild  2  3  4  5 =moderate  6  7  8  9  10 =very severe |
| *(If Q6 response of 1 or 2 then skip to Q8)* What specific aspects of COVID-19 are worrying you? | -- select all that apply -- | - Nothing - Social isolation - I have COVID-19 - Someone I know has COVD-19 - Not being able to go out as usual - I’ve lost my job or hours have been reduced - My friends or family have lost their job/s or hours have been reduced - Not knowing when the pandemic will end - Not knowing what the future will be like once the pandemic is over - Other: (open text) |
| Think about the strategies you normally use to feel better when you’re distressed*.* Do you think you have been using these strategies more, the same, or less since COVID-19? | -- select one -- | Check boxes for each of the 14 items:   - use more - use the same - use less - do not use   `Exercise  `Using social support  `Over-eating/comfort food  `Self-harm  `Sleeping too much  `Relaxation techniques  `Mindfulness meditation  `Spending too much money  `Drug and/or alcohol use  `Social Withdrawal  `Avoidance/procrastination  `Seeking professional help (face-to-face or online)  `Journaling  `Engaging in hobbies |
| In general, do you think these strategies have been less effective during COVID-19? | -- select one -- | 1=not at all  2=a little  3=some of the time  4= a lot  5=all of the time |
| Have you noticed any symptoms improve during, or following, the COVID-19 pandemic? | -- select all that apply -- | - No, none of my symptoms have improved - Yes, my anxiety symptoms have improved - Yes, my depression symptoms have improved - Yes, the frequency or severity of my suicidal thoughts have improved - I have noticed other improvements to my mental health: (open text) |

| **Loneliness (Adapted from The UCLA Three item Loneliness Scale)^11^** | | | |
| --- | --- | --- | --- |
| **The next questions are about how you feel about different aspects of your life during the COVID-19 social restrictions. For each one, tell me how often you feel that way.** | **Hardly Ever** | **Some of the Time** | **Often** |
| I feel I lack companionship | 1 | 2 | 3 |
| I feel left out | 1 | 2 | 3 |
| I feel isolated | 1 | 2 | 3 |
| I have meaningful social contact | 1 | 2 | 3 |

**Extreme weather/climate events**

| **Extreme weather/climate events (created for this study)**  **Recently Australia has experienced a series of extreme weather events, including bushfires in the 2020 New Year’s period followed by floods. Some regions of Australia have also experienced drought over the past few years. We’d just like to ask you about extreme weather/climate events you may have experienced.** | |
| --- | --- |
| Question | Response options |
| In the past 12 months, have you personally experienced any of the following extreme weather events? Check all that apply: | Drought  Bushfire  Flood  Cyclone  Other – please specify:  No, I have not experienced any of these events |
| Have you lived in a region that has been directly affected by any of the following extreme weather events in the past 12 months? Check all that apply: | Drought  Bushfire  Flood  Cyclone  Other – please specify:  No, I have not experienced any of these events |
| If you have experienced more than one of these events, which one had the greatest health, mental health, or economic impact on you? | Drought  Bushfire  Flood  Cyclone  Other – please specify: |
| *If yes to either question above then continue:*  Please indicate how you were negatively affected as a direct result of the extreme weather event/s. Check all that apply. | My property was damaged/ destroyed  I had to relocate  I was unable to work or had my work hours reduced  I lost my job  I could not access health services when I needed them  I became injured  A family member became injured  A family member died  Someone close to me was injured or died  I experienced anxiety for the first time  I experienced depression for the first time  I experienced suicidal thoughts for the first time  I began to self-harm (injure self without suicidal intent)  I had increased problems in personal relationships  I had/have increased anxiety and/or stress levels  I had/have experienced increased depression levels  I had/am experiencing suicidal thoughts  I had/am experiencing increased self-harm  I have attempted suicide |
| Have any of the problems which you have identified been amplified or made worse by the coronavirus (COVID-19) pandemic? | Yes  No  Unsure |
| If yes then: Which of the problems are **worse** as a result of COVID-19? Check all that apply. | My property was damaged/ destroyed  I had to relocate  I was unable to work or had my work hours reduced  I lost my job  I could not access health services when I needed them  I became injured  A family member became injured  A family member died  Someone close to me was injured or died  I had increased problems in personal relationships  Anxiety symptoms  Depression symptoms  Suicidal thoughts  Self harm (non-suicidal intent)  Suicidal behaviour |

| **Social Determinants (Created for this survey)** | |
| --- | --- |
| **Question** | **Response Options** |
| Please describe your economic situation. Check all that apply. | My economic situation is secure, and will remain secure for the foreseeable future  My economic situation has been negatively impacted due to COVID-19, but is still secure for the foreseeable future  My economic situation has been severely negatively impacted due to COVID-19 and may deteriorate if the situation persists  My economic situation has been severely negatively impacted due to COVID-19 and is in immediate decline |
| How are you engaged with your community? Check all that apply. | Volunteering  Committee participation in community events  Attending community organized events  Social gatherings  Member of a sports, scouting, or other club  Other  None |
| Please describe your current housing situation. Check all that apply. | I own a property  I rent a property  Secure, and unlikely to change in the near future  Secure, but may change in the near future due to COVID-19  Not secure and likely to change in the near future due to COVID-19  Uncertain due to COVID-19 |
| Do you have a chronic or acute illness/es that require treatment or medication? | Yes  No |
| If ‘yes’ then: Do you have the means and access to the necessary treatment/ medication? | Yes  No |
| Do you have private medical insurance? | Yes  No |
| If the need arose, how confident are you that you would be able to access necessary medical care? | Not confident at all  Not very confident  Somewhat confident  Very confident  Completely confident |
| In general, how often is there conflict, stress, or tension between members of your household? Check all that apply. | I live alone Never, it’s very peaceful  A little  Some of the time  A lot  All of the time |

| **Extended E-HITS (Hurt, Insult, Threaten, Scream) Screening Tool for detecting IPV^12^ (Only for participants aged 18 and above)**  **Please indicate how often your partner did each of these things in the past month.** | |
| --- | --- |
| Question | Response options |
| Has your partner ever physically hurt you in the past month? | 1. Never  2. Rarely  3. Sometimes  4. Often  5. Frequently |
| Has your partner ever insulted you in the past month? | 1. Never  2. Rarely  3. Sometimes  4. Often  5. Frequently |
| Has your partner ever threatened to harm you in the past month? | 1. Never  2. Rarely  3. Sometimes  4. Often  5. Frequently |
| Has your partner ever screamed or cursed at you in the past month? | 1. Never  2. Rarely  3. Sometimes  4. Often  5. Frequently |
| Has your partner ever forced you into sexual activities in the past month? | 1. Never  2. Rarely  3. Sometimes  4. Often  5. Frequently |

**Survey References**

1. van Spijker, B. A., Batterham, P. J., Calear, A. L., Farrer, L., Christensen, H., Reynolds, J., & Kerkhof, A. J. (2014). The suicidal ideation attributes scale (SIDAS): Community-based validation study of a new scale for the measurement of suicidal ideation. Suicide and Life-Threatening Behavior, 44(4), 408-419. doi:10.1111/sltb.12084
2. Kroenke, K., Spitzer, R. L., & Williams, J. B. (2001). The PHQ-9: validity of a brief depression severity measure. Journal of General Internal Medicine, 16(9), 606-613.
3. Spitzer, R. L., Kroenke, K., Williams, J. B., & Lowe, B. (2006). A brief measure for assessing generalized anxiety disorder: the GAD-7. Archives of Internal Medicine, 166(10), 1092-1097.
4. Batterham, P. J., Sunderland, M., Carragher, N., Calear, A. L., Mackinnon, A. J., & Slade, T. (2016). The Distress Questionnaire-5: Population screener for psychological distress was more accurate than the K6/K10. Journal of Clinical Epidemiology, 71, 35-42. doi:10.1016/j.jclinepi.2015.10.005
5. Duckworth, A. L., Peterson, C., Matthews, M. D., & Kelly, D. R. (2007). Grit: perseverance and passion for long-term goals. *Journal of Personality and Social Psychology, 92*(6), 1087-1101. doi:10.1037/0022-3514.92.6.1087
6. Duckworth, A. L., & Quinn, P. D. (2009). Development and Validation of the Short Grit Scale (Grit–S). *Journal of Personality Assessment, 91*(2), 166-174.
7. McNeely, J., Wu, L. T., Subramaniam, G., Sharma, G., Cathers, L. A., Svikis, D., Sleiter, L., Russell, L., Nordeck, C., Sharma, A., O'Grady, K. E., Bouk, L. B., Cushing, C., King, J., Wahle, A., & Schwartz, R. P. (2016). Performance of the Tobacco, Alcohol, Prescription Medication, and Other Substance Use (TAPS) Tool for Substance Use Screening in Primary Care Patients. Annals of internal medicine, 165(10), 690–699. <https://doi.org/10.7326/M16-0317>
8. Rickwood, D. J., & Braithwaite, V. A. (1994). Social-psychological factors affecting help-seeking for emotional problems. Soc Sci Med, 39(4), 563-572.Wilson, C. J., Deane, F. P., Ciarrochi, J., & Rickwood, D. (2005). Measuring Help-Seeking Intentions: Properties of the General Help-Seeking Questionnaire. Canadian Journal of Counselling, 39(1), 15-28
9. Wilson, C. J., Deane, F. P., Ciarrochi, J., & Rickwood, D. (2005). Measuring Help-Seeking Intentions: Properties of the General Help-Seeking Questionnaire. Canadian Journal of Counselling, 39(1), 15-28.
10. Sarason, I. G., Sarason, B. R., Shearin, E. N., Pierce, G. R. (1987). A brief measure of social support: Practical and theoretical implications. Journal of Social and Personal Relationships, 4, 497-510.
11. Hughes, M. E., Waite, L. J., Hawkley, L. C., & Cacioppo, J. T. (2004). A Short Scale for Measuring Loneliness in Large Surveys: Results From Two Population-Based Studies. Research on Aging, 26(6), 655-672. doi:10.1177/0164027504268574
12. Chan, C. C., Chan, Y. C., Au, A., & Cheung, G. O. C. (2010). Reliability and validity of the “Extended-Hurt, Insult, Threaten, Scream”(E-HITS) screening tool in detecting intimate partner violence in hospital emergency departments in Hong Kong. Hong Kong Journal of Emergency Medicine, 17(2), 109-117.
